# Demographic Outcomes and Ecosystem Implications of Giant Tortoise Reintroduction to Española Island, Galapagos

**DOI:** 10.1371/journal.pone.0110742

**Published:** 2014-10-28

**Authors:** James P. Gibbs, Elizabeth A. Hunter, Kevin T. Shoemaker, Washington H. Tapia, Linda J. Cayot

**Affiliations:** 1 Department of Environmental and Forest Biology, State University of New York College of Environmental Science and Forestry, Syracuse, New York, United States of America; 2 Warnell School of Forestry and Natural Resources, University of Georgia, Athens, Georgia, United States of America; 3 Department of Ecology and Evolution, Stony Brook University, Stony Brook, New York, United States of America; 4 Department of Applied Research, Galapagos National Park Service, Puerto Ayora, Galapagos, Ecuador; 5 Galapagos Conservancy, Fairfax, Virginia, United States of America; The Biodesign Institute, Arizona State University, United States of America

## Abstract

Restoration of extirpated species via captive breeding has typically relied on population viability as the primary criterion for evaluating success. This criterion is inadequate when species reintroduction is undertaken to restore ecological functions and interactions. Herein we report on the demographic and ecological outcomes of a five-decade-long population restoration program for a critically endangered species of “ecosystem engineer”: the endemic Española giant Galapagos tortoise (*Chelonoidis hoodensis*). Our analysis of complementary datasets on tortoise demography and movement, tortoise-plant interactions and Española Island’s vegetation history indicated that the repatriated tortoise population is secure from a strictly demographic perspective: about half of tortoises released on the island since 1975 were still alive in 2007, *in situ* reproduction is now significant, and future extinction risk is low with or without continued repatriation. Declining survival rates, somatic growth rates, and body condition of repatriates suggests, however, that resources for continued population growth are increasingly limited. Soil stable carbon isotope analyses indicated a pronounced shift toward woody plants in the recent history of the island’s plant community, likely a legacy of changes in competitive relations between woody and herbaceous plants induced by now-eradicated feral goats and prolonged absence of tortoises. Woody plants are of concern because they block tortoise movement and hinder recruitment of cactus–a critical resource for tortoises. Tortoises restrict themselves to remnant cactus patches and areas of low woody plant density in the center of the island despite an apparent capacity to colonize a far greater range, likely because of a lack of cactus elsewhere on the island. We conclude that ecosystem-level criteria for success of species reintroduction efforts take much longer to achieve than population-level criteria; moreover, reinstatement of endangered species as fully functioning ecosystem engineers may often require large-scale habitat restoration efforts in concert with population restoration.

## Introduction

Restoration of extirpated species and populations via captive breeding and reintroduction has become a widespread practice in biodiversity conservation [1]–[2]. As such projects proliferate there is growing need to define and document reintroduction success [2]–[3]. Documentation of a viable, self-sustaining population is essential for declaring success of any reintroduction project [4]. Yet reintroduction efforts are often motivated not just by population viability considerations but also by broader concerns about restoring ecological functions and interactions, thereby highlighting the need to document the role of reintroduced species as agents of ecosystem change [4].

Species reintroduction can generate profound and complex ecosystem changes, especially if the focal species can be characterized as an “ecosystem engineer” [5]–[7]. For example, carnivore reintroduction to Yellowstone National Park in the western United States likely triggered a trophic cascade whereby predation pressure on large herbivores has led to the restoration of plant communities [6],[8]. Yet ecosystem-level effects of species reintroduction may manifest only after the focal species crosses a threshold of effective density far higher than needed to ensure population persistence alone [9]–[10] and thereby result in separate time frames and planning horizons for achieving population-level versus ecosystem-level objectives of species reintroduction programs.

Despite the promise of species reintroduction as a tool for restoring populations and in some cases ecosystems, prolonged absence of certain species can result in habitat degradation and thereby lead to failed reintroduction efforts despite repeated attempts [1],[4],[11]–[12]. In some cases, management interventions may be required to render release sites hospitable to a focal species before the species can assume its former ecological role [13]. For example, invasive species removal may facilitate successful repatriation in cases where a species’ former ecological role has been supplanted by an invasive competitor [14]. For these reasons assessments of the success and feasibility of reintroduction projects must carefully consider ecological changes wrought in the absence of the focal species.

Reintroduction of tortoise populations is increasingly proposed not only to conserve biodiversity [15] but also as tools for ecosystem restoration [16]–[18]. Giant tortoises, once widespread on all continents except Antarctica, are ecosystem engineers that manipulate the distribution and abundance of other organisms through direct effects of herbivory, disturbance and seed dispersal on plant communities and subsequent indirect impacts on animal communities [14],[19]–[20]. The effects of giant tortoises on terrestrial ecosystems of oceanic islands (to which giant tortoises are currently restricted) are potentially on par with those of megaherbivores as drivers of savanna structure and function, a system for which the role of ecosystem engineers as agents of ecosystem change is far better understood [21]–[22]. Consequently, giant tortoises have been proposed as generic tools for island ecosystem restoration [17].

**Figure 1:**
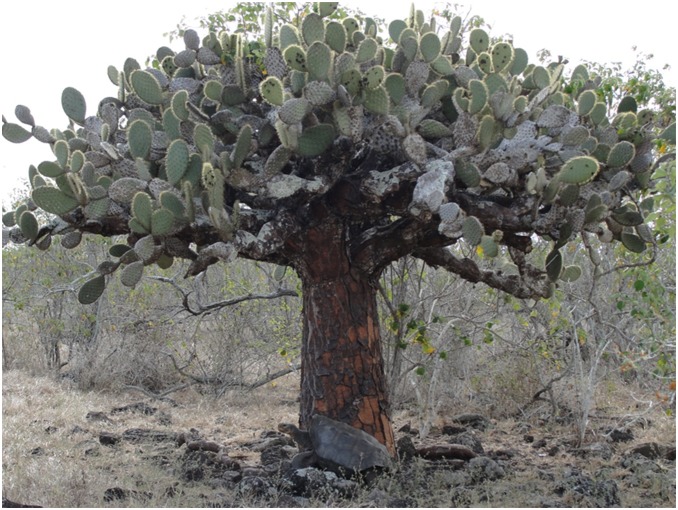
Adult male Galapagos giant tortoise (*Chelonoidis hoodensis*) resting beneath an adult arboreal prickly pear cactus (*Opuntia megasperma*) surrounding by woody plants (mostly muyuyo or *Cordia lutea*), Española Island, May 2010 (image: J. P. Gibbs).

Herein we examine the demographic and ecological outcomes of the repatriation of Galapagos giant tortoises (*Chelonoidis hoodensis*) to Española Island (Fig. 1). *C. hoodensis* is a CITES Appendix I and IUCN Red List “critically endangered” species [23] that once numbered in the thousands of individuals but dropped to just 15 individuals by 1960 due to historical exploitation of tortoises throughout the archipelago [24]. Between 1963 and 1974, all survivors (12 females and 3 males) were brought into captivity and to date >1500 of their captive-raised offspring have since been released onto the island [25]. Successful reproduction of repatriated *C. hoodensis* was first documented in 1990 [24] suggesting the repatriated population might become self-sustaining.

Concerns remain, however, about current and future habitat quality. Specifically, the island’s dense and extensive woody plant cover is hypothesized to inhibit tortoise movement (thereby excluding tortoises from colonizing new areas) and to outcompete grasses and juvenile cactus that are critical to tortoises as forage [16]. The present-day dominance of woody plants on Española Island may be a legacy of both the absence of giant tortoises and the presence of large numbers of feral goats on the island for ¾ of a century (1905 to 1978), which might have shifted competitive relationships between woody and herbaceous plants by altering soil water availability in ways that favor woody plants [26]–[28]. Furthermore, sudden eradication of introduced livestock (in this case, goats) in the absence of native herbivores (in this case, giant tortoises) may have exacerbated woody plant incursion [28]. A particular concern relates to tortoise interactions with a large-seeded, arboreal prickly pear cactus (*Opuntia megasperma*). The cactus is restricted to just three islands and is listed by the IUCN as a “vulnerable” species due to extensive population reduction caused by feral goats. The cactus serves as a critical source of food, water, and shade for tortoises [29]. Although tortoises have been observed to injure and crush young *O. megasperma* individuals, in aggregate tortoise impacts on cactus populations, including seed dispersal [20], appear to be marginally positive on Española Island [16].

We integrated complementary datasets on tortoise demography, tortoise-plant interactions and vegetation history on Española Island to comprehensively assess the reintroduced tortoise population’s trajectory in the context of past, current and future ecological conditions. We first examined a 32-year-long tortoise mark-recapture dataset for evidence that the population is self-sustaining. We then assessed the relationship between tortoise density and habitat features (including cactus and woody plant density) in the context of historical changes in woody plant cover on the island. Last, we simulated how different management scenarios, including cessation of the repatriation program and propagation of cactus and control of woody plants, might affect the long-term stability of the tortoise population. We demonstrate that the first step of restoring a viable population of giant tortoises has been achieved; however, significant habitat restoration challenges remain before the role of tortoises as self-sustaining, island-wide ecosystem engineers is fully realized.

## Materials and Methods

### Study area and the tortoise monitoring program

**Figure 2:**
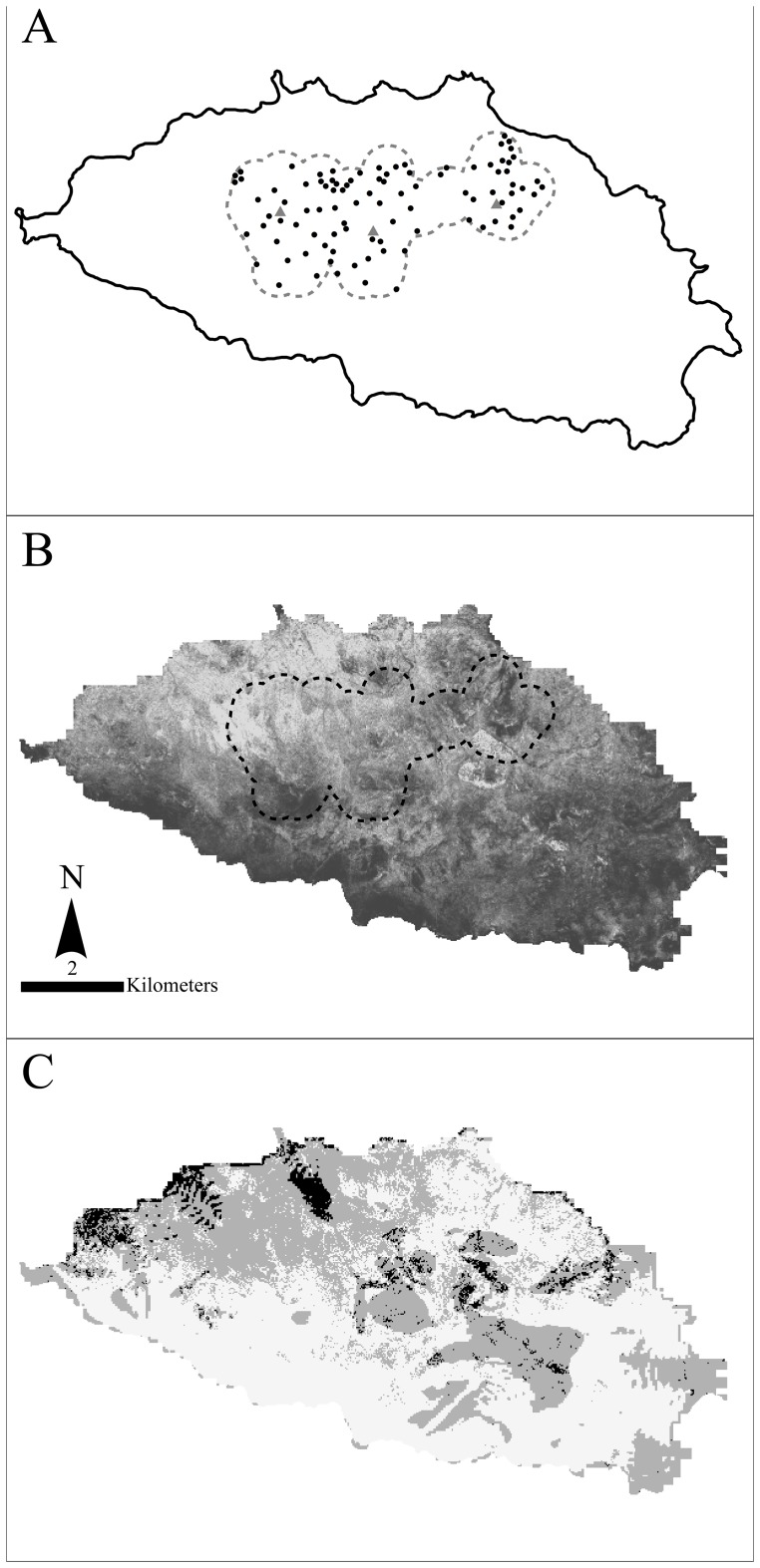
Study areas on Española Island. (A): gray dashed line is “tortoise zone” or maximum extent of tortoises observed on the island since the repatriation program was initiated; black circles are 2010 survey plot locations for tortoises, cactus, and woody plants; gray triangles are tortoise introduction sites (West: Tunas, central: Cacos, East: Gardner). Woody plant cover on Española Island (B; darker shades indicate higher percent woody plant cover), classified using cloud-free Quickbird satellite imagery (December 29, 2006, 0.6 meters resolution) into areas with and without woody vegetation using a supervised classification calibrated with plot woody plant density estimates (IDRISI Andes 5.0) (percent cover is the percent vegetated cells per 24 m×24 m area). Potentially highly suitable cactus restoration areas (C) with low slopes (<2.06 degrees, bottom quartile of available slopes) and low woody plant cover (<52% cover woody plants, bottom quartile of available woody plant cover); black areas have both low slopes and low woody cover, gray areas have either low slope or low woody cover.

Española Island (−1° 38′ N; −89° 67′ W) occupies the southeastern sector of the Galapagos Archipelago and is small (60.48 km^2^, <1% of the archipelago), low elevation (max. 206 m), flat, and arid (annual precipitation varies between 10–600 mm with average annual temperatures of 23.8°C). Dominant herbaceous plants include *Galactia striata* and *G. tenuiflora, Phaseolus mollis*, *Rhynchosia minima* and *Paspalum* spp. Woody plants, primarily *Cordia lutea* and *Prosopis juliflora*, occur at high densities throughout the island (Fig. 2). The arboreal *Opuntia* cactus is almost entirely restricted to the island’s central region [30], a 1250-hectare area that encompasses the three sites used for releasing repatriates from the *ex situ* breeding program: El Caco, Las Tunas, and Gardner Bay (Fig. 2a). Releases began with 17 captive-bred tortoises in 1975 and continued with 2- to 5-year-old tortoises introduced every year from 1975–1994 and sporadically thereafter from 1997–2007 for a total of 1482 tortoises repatriated [29].

Since the reintroduction program was initiated all repatriated tortoises as well as individuals encountered in the field during surveys have been given unique identification markings. Up to 1991, a unique combination of notches (made with machete or metal file) was carved onto the tortoises’ marginal scutes. Thereafter a duplicate marking system was introduced consisting of numbers branded on the rear costal scutes combined with insertion of passive integrated transponder (PIT) tags (AVID Identification Systems Inc., Norco, California, USA) into the left rear leg of each tortoise. Locations of individual tortoises have been registered since 2002 with hand-held Global Positioning System (GPS) devices, thereby enabling tracking movements of marked individuals between survey bouts during later phases of the tortoise monitoring program. Most (96%) tortoises captured were weighed with a calibrated spring scale. Curved carapace length, measured as the distance from the notch at the anterior of the carapace over the tops of the vertebral scutes to the tip of the supracaudal scute, was used as the measure of tortoise size. Sex and age were assessed on the basis of external morphology. Native-hatched tortoises (offspring of repatriates), first encountered in significant numbers after 2000, were identified on the basis of lack of notches or PIT tags and the distinct appearance of their scutes: more compressed growth segments, lighter coloration, and more scute abrasion given size.

Fieldwork was carried out on an endangered species and on public land in strict accordance with permit conditions of the Galapagos National Park Directorate and following the protocol approved by State University of New York, College of Environmental Science and Forestry’s Institutional Animal Care & Use Committee (Protocol Number: 121201).

### Tortoise-plant interactions

Because of the hypothesized importance of woody plants as a potential factor structuring tortoise-plant interactions and thereby affecting tortoise population recovery, we quantified the extent of woody vegetation on Española Island over both space and time. Spatial extent was determined by delineating woody vegetation from cloud-free Quickbird satellite imagery (December 29, 2006, 0.6 meters resolution) using a supervised classification calibrated with woody plant density estimates from field plots (see below, IDRISI Andes 5.0). We aggregated these data to obtain a map of percent woody cover by summing the number of 0.6×0.6 m cells classified as woody in each 24 m cell. Temporal trend in woody plant cover, emphasizing possible changes associated with goat impacts and tortoise near-absence over the last 100 years, was inferred from soil carbon isotope analysis [31]. To do so, we first determined diagnostic δ^13^C signatures of the island’s dominant woody plants (*Bursera*, *Cordia*, *Croton*, *Parkinsonia*, *Prosopis*) and herbaceous plants (*Cyperus* and *Paspalum*) as well as cactus (*Opuntia*) (*n* = 10 of each species). We then sampled soil at 10 cm increments through the soil horizon from soil pits dug at Gardner Bay and in the Caco area. All analyses were made at the Environmental Stable Isotope Laboratory at SUNY-ESF in Syracuse, New York. Accuracy and precision of stable isotope measurements were verified using National Institute of Standards and Technology (NIST) reference materials (M. A. Teece, pers. comm.). Radiocarbon dating of two soil samples at two points in the soil profile of the Caco pit was performed by Beta Analytic Inc., Miami, Florida, to enable calibration of soil depth and soil sample age. We tested trends in the dominance of woody plants in the soil record using linear models of δ^13^C by depth for two time periods (recent history versus earlier, see Results) using a general linear mixed model with soil pit treated as a random effect.

To assess specific hypotheses about tortoise-plant interactions we performed two studies. The first was re-censusing cactus in June 2014 in a 60 ha macroplot in the Tunas tortoise release area first censused in May 2004 [16] to examine cactus population trends in an area where tortoises have long been established at high density. The second involved measuring tortoise and cactus abundance as well as woody vegetation extent on 96 field plots within the central “tortoise zone” in May-June 2010. The sampling frame was established by buffering by 250 m all tortoise occurrences recorded during previous surveys. We then randomly placed one plot within every 250×250 m grid cell. Each plot comprised five 20-m-radius circular sub-plots with a central sub-plot and four sub-plots directly adjacent at the cardinal directions. Within each sub-plot we recorded total numbers of tortoises, tortoise droppings, juvenile cactus (no bark on trunk), subadult cactus (some bark on trunk), and adult cactus (trunk entirely bark-covered). Density of woody plants was estimated using the point-quarter method [32] for two basal diameter classes: ≥5 cm (mature plants) and≤0.5 cm (recruits). All measurements were subsequently averaged at the plot-level for analysis of interactions among tortoises, cactus, and woody vegetation via binary classification trees [33]. We also modeled tortoise density as a function of cactus and woody plant density, as well as distance from release site, using a linear negative binomial regression model. The model was run in WinBUGS, using 30,000 MCMC iterations (treating the first 10% as a burn-in and capturing every 10^th^ observation to reduce serial autocorrelation).

After 30 years of repatriation, tortoises remain restricted to the central part of the island (Fig. 2a); therefore, we evaluated whether tortoises have the intrinsic capacity to move further afield if suitable habitat were available. To do so, observed annual displacement rates (km per year) of individuals from 2000 to 2003 were used as a proxy for intrinsic capacity for range expansion. We tested for differences among age/sex classes (juvenile and adult, female and male) in mean displacement rates with a linear mixed effects model and then examined whether the observed expansion in range area (computed as a minimum convex polygon, or MCP, over all known occurrence locations) differed from a null expectation associated with random movement. We generated the null distribution for range expansion by simulating 500 replicate movement scenarios in which observed starting locations were randomly permuted (thereby de-linking starting locations from observed movement distances and directions). Finally, we tested the hypothesis that tortoises near the edge of the tortoise zone would be more likely to move away from the current range than tortoises situated far from the edge by regressing the direction of tortoise movement relative to the nearest edge with the distance of the starting location from the nearest edge. All analyses were run in the R statistical computing language (raster package and adehabitatHR package) [35]–[36].

Last, to estimate carrying capacity (K) for tortoises of the central zone they now occupy (Fig. 2a), we used the regression model of tortoise density (described previously) to compute the expected tortoise density at release sites, extrapolating the resulting estimate to the rest of the tortoise zone (assuming tortoises had reached carrying capacity at distance = 0 from release sites and that mean cactus and woody plant densities computed from plot-level data pertained to the entire tortoise zone). In addition, we used the same model to evaluate island-wide carrying capacity under three potential restoration scenarios: (1) a cactus regeneration scenario in which the cactus density of the tortoise zone is restored to the entire island, (2) a woody plant removal scenario in which woody plant density is reduced by half across the entire island, and (3) a combined cactus regeneration/woody plant removal scenario. The potential effect of these restoration scenarios was expressed as the percent increase in the tortoise carrying capacity over the current estimated carrying capacity.

### Tortoise demography

We assessed the demographic outcome of the tortoise repatriation program in two ways. The first was direct estimation of tortoise population size in 2010 (after 34 years of repatriations) from mean tortoise densities measured on the 96 field plots in the tortoise zone (see above). The second involved estimation of abundance, vital rates, growth rates and body condition based on a capture history database assembled from surveys between 1975 and 2007 (recapture data were available every year except 1995, 1996 and 1998), during which an average of 120 tortoises was recaptured per year (range: 16 to 377). Trends in somatic growth (cm/year) and body condition (deviations from expected mass-length relationships) were assessed using generalized additive mixed models (GAMMs, mgcv [34]) that included the categorical covariates sex/age (juvenile, adult, male, female or unknown) and individual (as a random effect in order to account for repeated measures [37]) as well as year (1975–2007) and body size (curved carapace length). Growth was determined from change in curved carapace length between subsequent measurements of tortoises (2.5% percent of observations were removed from both extremes of the growth rate estimates distribution as well as recaptures >3 years apart to guard against anomalies caused by errors in field recording or data entry).

We also extracted annual abundance and survival rates from the capture history database using a Bayesian open-population model (Cormack-Jolly-Seber analogue) in conjunction with the known numbers of tortoises released each year. We estimated survival (φ) separately for juveniles (<8 y.o.) and subadults/adults (≥8 y.o.). To accommodate temporal dynamics in survival rate (e.g., environmental stochasticity, trends in survival, density dependence), survival rates were estimated separately for each 4-year time period (subadults/adults), 2-year time periods (juveniles), and 1-year time period (year-of-release). Mean capture probability was estimated separately for juveniles/subadults (<15 y.o.) and reproductive adults (≥15 y.o.) with inter-annual variance in capture probability modeled as a logit-normal random effect. Because our modeling framework explicitly tracked the status of each released tortoise (i.e., alive or dead) each year, we were able to estimate annual abundance of released tortoises (excluding native-born individuals) at two distinct release areas (Tunas and Caco versus Gardner Bay) as the sum of living individuals occupying each of these release areas (Fig. 2a) (due to proximity and lack of barriers to movement of tortoises between the release sites of El Caco and Las Tunas these were treated as a single release site for analysis).

We estimated population parameters with Markov-Chain Monte Carlo (MCMC) in WinBUGS (version 1.4) via the R2WinBUGS package in R [34]. Uninformative uniform prior probability distributions were assigned to all parameters and the initial 5,000 MCMC samples were discarded as a burn-in. We performed a further 10,000 MCMC iterations and saved every 10^th^ iteration to reduce serial autocorrelation among samples [38]. Convergence of the Markov chains to the stationary posterior distribution was tested with the Gelman–Rubin diagnostic [38]. We summarized posterior distributions for all parameters with the mean of all MCMC samples as a point estimate and the 2.5 and 97.5 percentiles of the MCMC samples as a 95% credible interval [38]. A detailed description of our demographic and population projection model is provided in Appendix S1 with accompanying R and WinBUGS code in Appendix S2.

We projected over the next 100 years the abundance of giant tortoises on Española Island on the basis of estimates of survival and temporal variation in survival derived from the capture-recapture analysis, carrying capacity estimates from tortoise-habitat relationships (see above), and fecundity estimates extrapolated from observations of native-born tortoises (see Appendix S3). We used a female-only model, because females are generally presumed to be the limiting sex for most turtles and tortoises [39]. Survival rates of tortoises aged 1–2 were not estimable (all tortoises were released at age 3 and above) and were assigned a range of values typical of long-lived turtle species [40]. Initial female abundance was approximated using the estimated total number of released individuals that were alive in 2007 (derived from capture-recapture analyses, and ignoring native-born individuals). We used a “ceiling” type density dependence model in which the maximum population growth rate (R_max_) was defined by the vital rates estimated for this study (a reasonable assumption given the population was likely below carrying capacity for much of the study period). All parameter values used in our population models, and the information sources for these parameter values, are presented in Table 1.

**Table 1:** Summary of input parameters used to simulate the population dynamics of Española giant tortoises during and after repatriation to Española Island, Galapagos.

Parameter	Lower-UpperBound	Source
Age at firstreproduction	17–17	Age of oldest repatriated tortoises at year of first appearance of native-born individuals on the island [24]
Proportion ofpopulation female	0.3–0.7	Not known; wide interval centered on 0.5
Carrying capacity	424–3921	Estimated from tortoise density relationships to distance from introduction sites, and cactus and woody plant density (see Methods)
Fecundity	4–10	Mean annual eggs produced per female [68]
Annual survival,ages 1–3	0.6–0.9	Not known; estimated using rates typical for turtles [40] with wide interval to accommodate uncertainty
Hatchling survival(ages 0–1 years)	0.15–0.26	Estimated via simulation to obtain values of a fraction of native-born animals similar to that observed in the field
Mean hatchingsuccess rate	0.15–0.26	Estimated via simulation to obtain values of a fraction of native-born animals similar to observed in the field

Parameters estimated from the 32-year capture-recapture dataset include juvenile and adult survival, temporal variation in survival, and survival at year-of-release (see text).

Total fertility rate (total number of yearlings produced per reproductive adult) was computed as the product of four parameters: mean proportion of females in the population, mean number of eggs produced per female, mean hatching success rate, and mean neonate (hatchling) survival. We used outside sources of information to estimate the first two fertility components (Table 1) and an approximate-likelihood technique to estimate the remaining fertility parameters (see Appendix S1 for details): on the basis of the increase in the proportion of native-born individuals observed over time we concluded that the product of egg viability and hatchling survival on Española Island was between 0.023 (equivalent to both parameters set at 0.15) and 0.066 (equivalent to both parameters set at 0.26). Recruitment was modeled as a Poisson process [38]. Lacking information on the covariance structure of the four components of fertility, we conservatively assumed that all four parameters were perfectly correlated, which may underestimate population stability. All population projections consisted of 100 separate population simulations for 150 years each starting in the year 2011. For all simulations we recorded total abundance of tortoises and total abundance of adults (the ecological effects of tortoises are largely driven by adults; see below). Last, we estimated extirpation probability as the proportion of simulation runs reaching the quasi-extinction threshold (75 adults or fewer at any point during the simulation) after 150 years. All population projection models were coded in the R statistical computing language, and the code is provided in Appendix S3.

## Results

### Spatial and temporal pattern in woody plant extent on Española Island

**Figure 3:**
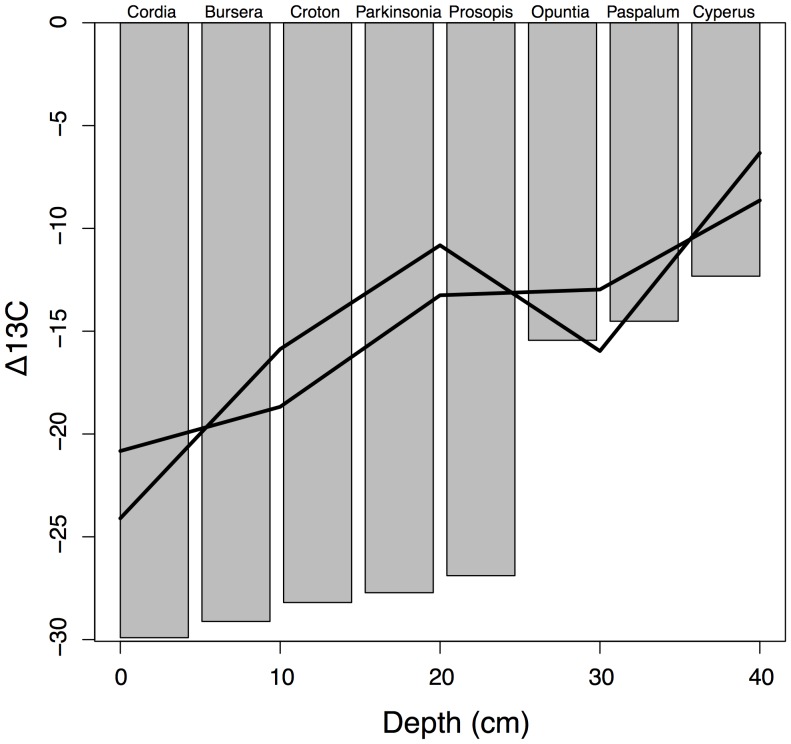
Carbon isotope signatures (δ^13^C values) of soils at increasingly greater depths in two soil pits (Gardner and El Caco) on Española Island, Galapagos. Radiocarbon dating indicated that depths of 40 cm are approximately 500 years BP; therefore, samples below the 40 cm depth are grouped to show pre-disturbance averages. Points are δ^13^C values of the island’s dominant woody plants (*Bursera, Cordia, Croton, Parkinsonia, Prosopis*) and herbaceous plants (*Cyperus* and *Paspalum*) as well as cactus (*Opuntia*) (averaged across 10 samples of each, with standard errors for all means too miniscule to be evident in this graphic).

Extent of woody plants on Española Island showed dramatic variation across the island in 2006 (Fig. 2b). Regression tree analysis indicated that the fraction of a given 24 m×24 m area on the island dominated by woody plants was highest (0.96) within 1.1 km of the coast; farther from the coast, woody cover was more extensive on lower slopes (<3.2 degrees, 0.86) than steeper ones and lower (0.58) in the tortoise zone than outside (0.68). In terms of temporal variation in woody cover, extent of woody plants apparently increased strongly and recently in terms of the history of the island’s plant community (Fig. 3). Radiocarbon dating from the Caco soil pit samples at 40 and 60 cm yielded dates of 510+/−40 BP and 1100+/−40 BP, respectively; thus our 0.8–1.1 m soil pits captured >1,000 years of vegetation history on the island. Stable carbon isotope signatures (δ^13^C values) were highly distinct between herbaceous and woody plants sampled: all woody plants were consistently more negative (−26 to −30‰) and all herbaceous plants consistently less negative (−13 to −16‰; Fig. 3) with cactus (−15‰) grouping among the complement of herbaceous plants sampled. In recent history (soil depths of ≦40 cm or within 510 YBP), δ^13^C values have become more negative with time (*t* = 5.49, *p*<0.001) indicating a shift towards greater dominance by woody plants (Fig. 3); prior to 510 YBP, δ^13^C values indicated no consistent trend (*t* = 0.38, *p* = 0.71).

### Tortoise-habitat relationships

**Figure 4:**
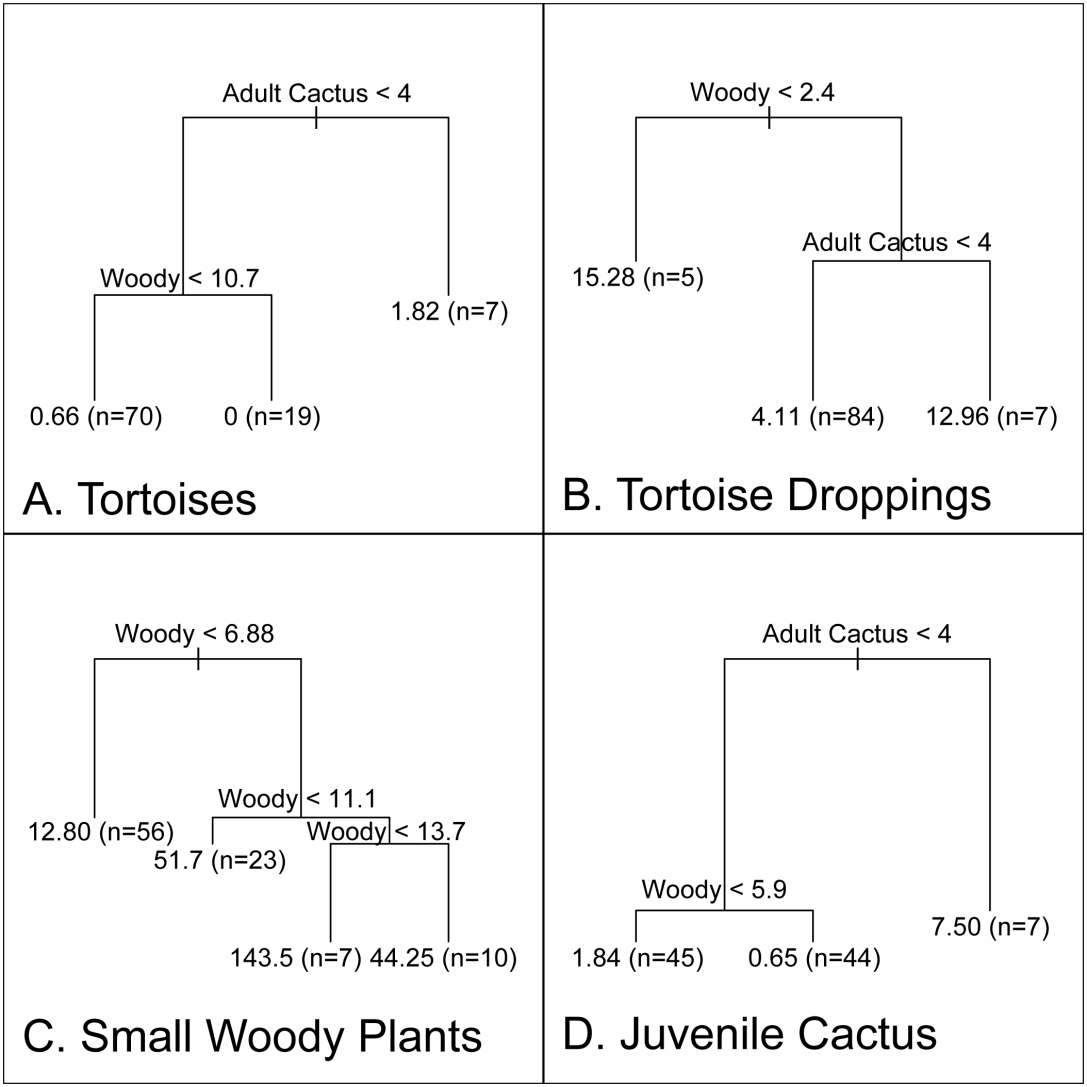
Relationships derived from binary classification trees between *density of tortoises* (A) and *tortoise droppings* (B) with thresholds of adult cactus density and large diameter woody plant density; between *small diameter woody plants* (C) with thresholds of large diameter woody plant density; and between *density of juvenile cactus* (D) with thresholds of large diameter woody plant density and adult cactus density. Density of tortoises, tortoise droppings, and cactus are expressed as number/hectare; woody plants as stems/100 m^2^.

In 2010 tortoise density was highest (1.8 tortoises/ha) in locations where adult cactus density was highest (>4.0 cactus/ha) (Fig. 4a). Where adult cactus density was lower (<4.0 cactus/ha), tortoises occurred at much lower densities (0.66/ha) and only if large diameter woody plant density was also low (<10.7 stems/100 m^2^). No tortoises occurred on plots with low adult cactus densities when combined with high densities of large diameter woody plants. Tortoise dropping density (reflecting tortoises in transit) was greatest (15.2/ha) where woody plant density was lowest (Fig. 4b) yet was still high (13.0/ha) in areas of high woody plant extent if adult cactus were present; otherwise there were dramatically fewer droppings (4.1/ha) suggesting that tortoises avoid woody plant areas particularly if no cactus is present.

Tortoise density in the tortoise zone declined with distance from reintroduction release points (Tunas, Caco and Gardner), increased with adult cactus density, and decreased with woody plant density. The credible interval of the effect of distance from introduction points did not overlap with zero (95% C.I.: −5.31 to −2.42), but it did for the cactus (95% C.I.: −0.14 to 2.33) and woody plant (95% C.I.: −2.45 to 2.04) effects. Using these relationships, we estimated a carrying capacity of 2081 tortoises (95% C.I.: 424 to 3921) within the tortoise zone. Under the three potential restoration scenarios, the change in the tortoise carrying capacity over the current carrying capacity was estimated to be a 7% increase under the cactus regeneration scenario, a 52% increase under the woody plant removal scenario, and a 62% increase under the cactus regeneration and woody plant removal scenario.

**Figure 5:**
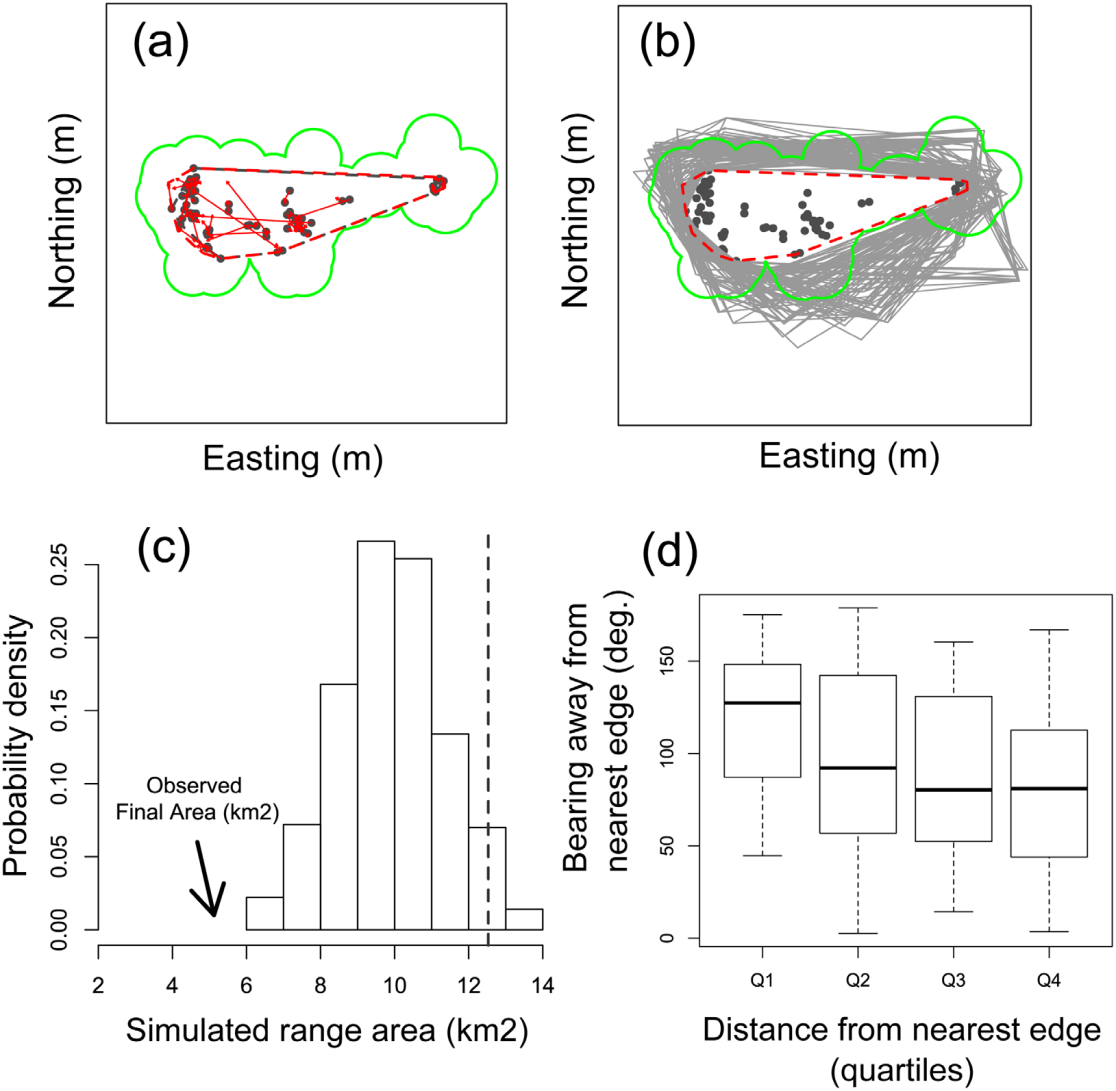
Movement patterns of giant tortoises reintroduced to Española Island, Galapagos. Closed circles in (A) and (B) represent the initial locations of all tortoises captured from 2000 to 2003 that were later recaptured. Arrows in (A) represent observed movement distances and directions. Minimum convex polygons (MCP) enclose the initial locations (grey) and initial plus final locations (red; differences represent the observed range expansion resulting from the observed movements). The grey polygons in (B) depict hypothetical MCPs (n = 500) for which initial locations have been decoupled from observed movement distances and directions. The observed final range area was smaller than all resulting hypothetical MCPs (C). In fact, those tortoises nearest to the range margins exhibited a strong tendency to move away from the nearest edge (D).

The area observed to be used by tortoises (MCP) expanded little during the period for which GPS locations were available (2000 and after; Fig. 5a). During this same period random movements by tortoises would have generated a substantial expansion of the currently occupied core area (Fig. 5b); in fact, the observed final MCP was entirely outside the statistical distribution of plausible final MCPs produced by random movement (Fig. 5c). This disparity suggests that tortoise movements were not random with respect to the edge of the observed core area, which was also confirmed by our final movement analysis: tortoises near to the edges of the core area were much more likely to move away from the current range edge than tortoises situated more centrally within the core area (Fig. 5d).

For cactus populations in the tortoise zone, we estimated densities of 1.7 juvenile cactus/ha, 0.4 subadult cactus/ha, and 1.0 adult cactus/ha for a total of 2142 juvenile cacti (95% C.I.: 1303.8–2980.3), 457.5 subadult cacti (201.7–713.2) and 1310.2 adult cacti (627.4–1992.9). Density of juvenile cactus was highest (7.5/ha) where adult cactus densities were >4 individuals/ha, next highest yet dramatically lower (1.8/ha) where adult cactus densities were <4 individuals/ha and density of large diameter woody plants was <5.9 stems/100 m^2^, and virtually absent (0.65/ha) where adult cactus reached densities of <4 individuals/ha and density of large diameter woody plants was >5.9 stems/stems/100 m^2^ (Fig. 4d). Density of small diameter woody plants (woody plant regeneration) was extremely high (143 stems/100 m^2^) at intermediate densities of large diameter woody plants (11–13 stems/100 m^2^) and not associated with tortoise density (Fig. 4c).

Over a 10-year period in the Tunas tortoise release zone macroplot [16], total individual cacti censused increased from 315 to 396 individuals (+26%): stage structure (adults versus juveniles and subadults) did not vary between census periods (G*_adj_* = 2.38, df = 1, P = 0.12) despite juveniles and subadults increasing in abundance (194 to 266 individual juveniles and subadults, +37%) more rapidly than adult cacti (121 to 130 adults, or +7%).

### Demographic reconstruction of the reintroduction program and future projections

Our recapture history comprised 1459 individuals from among cohorts initially released in El Caco (*n* = 780), Las Tunas (*n* = 370), and Gardner Bay (*n* = 309). Individuals were captured on average 2.2 times each (range: 1 to 21). Annual somatic growth rates (cm per year in curved carapace length) averaged 2.99 cm±2.19 cm (SD), with 35.6% of deviance explained by sex and age, carapace length, and year (Table 2): juveniles grew an average of 4.46 cm/year (±1.18 SD, n = 175), unknowns (intermediate between juveniles and adults) 3.23 cm/year (±2.19 SD, n = 361), males 2.25 cm/year (±2.27, n = 278) and females 2.31 cm/year (±2.01, n = 212). Our suite of independent variables accounted for virtually all variation (98%) in tortoise body mass: after controlling for the inherent size-mass relationship, males and unknowns were relatively lighter than females, and tortoise condition declined with time (Table 2).

**Table 2:** Summary of generalized additive mixed models fitted to giant tortoises (n = 909) on Española Island, Galapagos between 1975 and 2007 to explore relationships between (A) somatic growth rates (cm/year) and age/sex while controlling for time (year) and individual length (carapace length) (35.6% of deviance explained) and (B) body mass and age/sex while controlling for time (year) and individual length (carapace length) (97.8% of deviance explained).

(A) Somatic growth							
Parametric coefficients					Nonlinear effects (nonparametric)		
Parameter	Estimate	Standard Error	*t*	*P*	df	*F*	*P*
Constant	27.665	18.323	1.510	0.131			
Sex/Age (Female vs. Juvenile)	0.649	0.177	3.662	0.0003			
Sex/Age (Female vs. Male)	0.527	0.145	3.626	0.0003			
Sex/Age (Female vs. Unknown)	−0.209	0.172	−1.211	0.226			
Year	−0.0122	0.00918	−1.330	0.183			
Carapace length					5.466	49.550	0.001
**(B) Mass-length**							
**Parameter**	**Estimate**	**Standard Error**	***t***	***P***	**df**	***F***	***P***
Constant	173144.0	16114.663	10.745	0.001			
Sex/Age (Female vs. Juvenile)	29.967	162.393	0.184	0.853			
Sex/Age (Female vs. Male)	−447.683	144.858	−3.0912	0.00202			
Sex/Age (Female vs. Unknown)	−1190.300	175.237	−6.793	0.001			
Month							
Year	−78.221	8.073	−9.689	0.001			
Carapace length					8.992	8413	0.001

**Figure 6:**
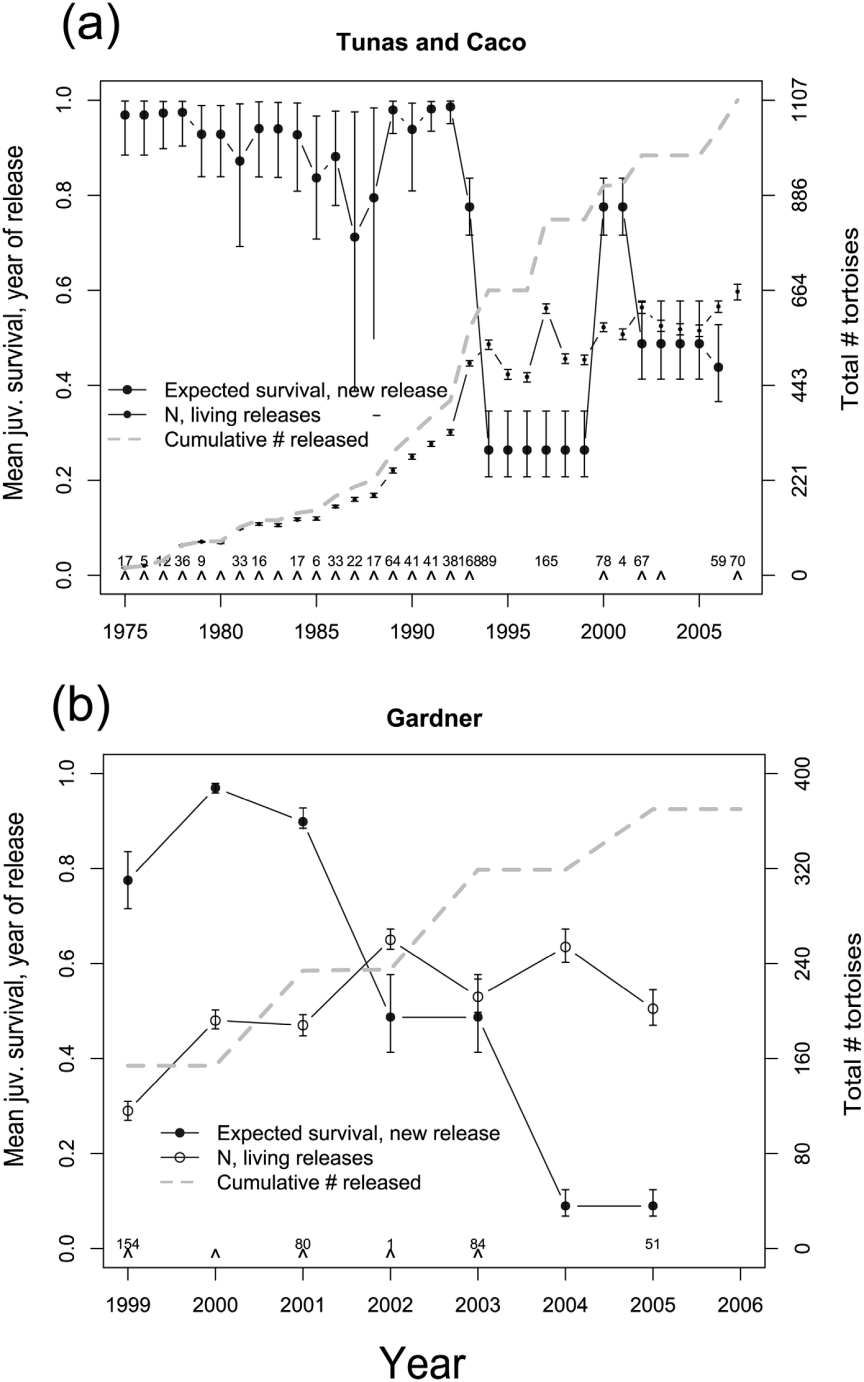
Estimates derived from a 32-year capture-recapture data set of abundance and vital rates of giant tortoises reintroduced to Española Island, Galapagos depicted by release sites (see Fig. 1): Tunas and Caco combined (A) and Gardner (B). Closed circles depict expected survival rates for newly released juveniles, the dashed line cumulative number of tortoises released, and open circles the estimated number of released individuals remaining. Release years are denoted with carets located above the x axis, and the numbers above the carets indicate the total number of tortoises released during each repatriation event.

Annual probability of capture for juvenile giant tortoises averaged 0.38 (CI 0.17–0.58), and was slightly lower for adult giant tortoises (Fig. S1–1 in Appendix S1). Mean annual survival was high for both juvenile (mean = 0.958, CI 0.86–0.99) and adult giant tortoises (mean = 0.977, CI 0.93–0.99), with mean juvenile survival approximately 1% lower than adult survival (Fig. S1-1 in Appendix S1). Adult survival rate varied temporally from 0.94 to 0.99, juvenile survival rate varied temporally from 0.88 to 0.99, and survival at year-of-release varied from 0.29 to 0.98, with generally lower survival rates after 1994 (Fig. 6a). Maximum population growth rate (R_max_, or the discrete population growth rate excluding density dependent effects) was estimated at 1.01 on the basis of our vital rate (survival and fecundity) estimates (popbio package in R [34]). With only 6 years of data for which to estimate survival and abundance at the “Gardner” release area, credible intervals for all survival terms were much more diffuse (Fig. 6b) and most survival information was derived from the much larger and longer-term records from the Tunas and Caco release site. We estimate that more than half of tortoises released to the island since 1975 were still alive in 2007 (Fig. 6a). Notably, the independent estimate of tortoise population size in 2010 derived from plot-based counts of tortoises for the Tunas and Caco area of 0.613 tortoises/ha (or total population of 769.5 tortoises, 439.7–1099.2, 95% confidence limits) corresponded well to the population size estimate derived from the mark-recapture analysis: 864 tortoises surviving in 2007 (Fig. 6a & b).

**Figure 7:**
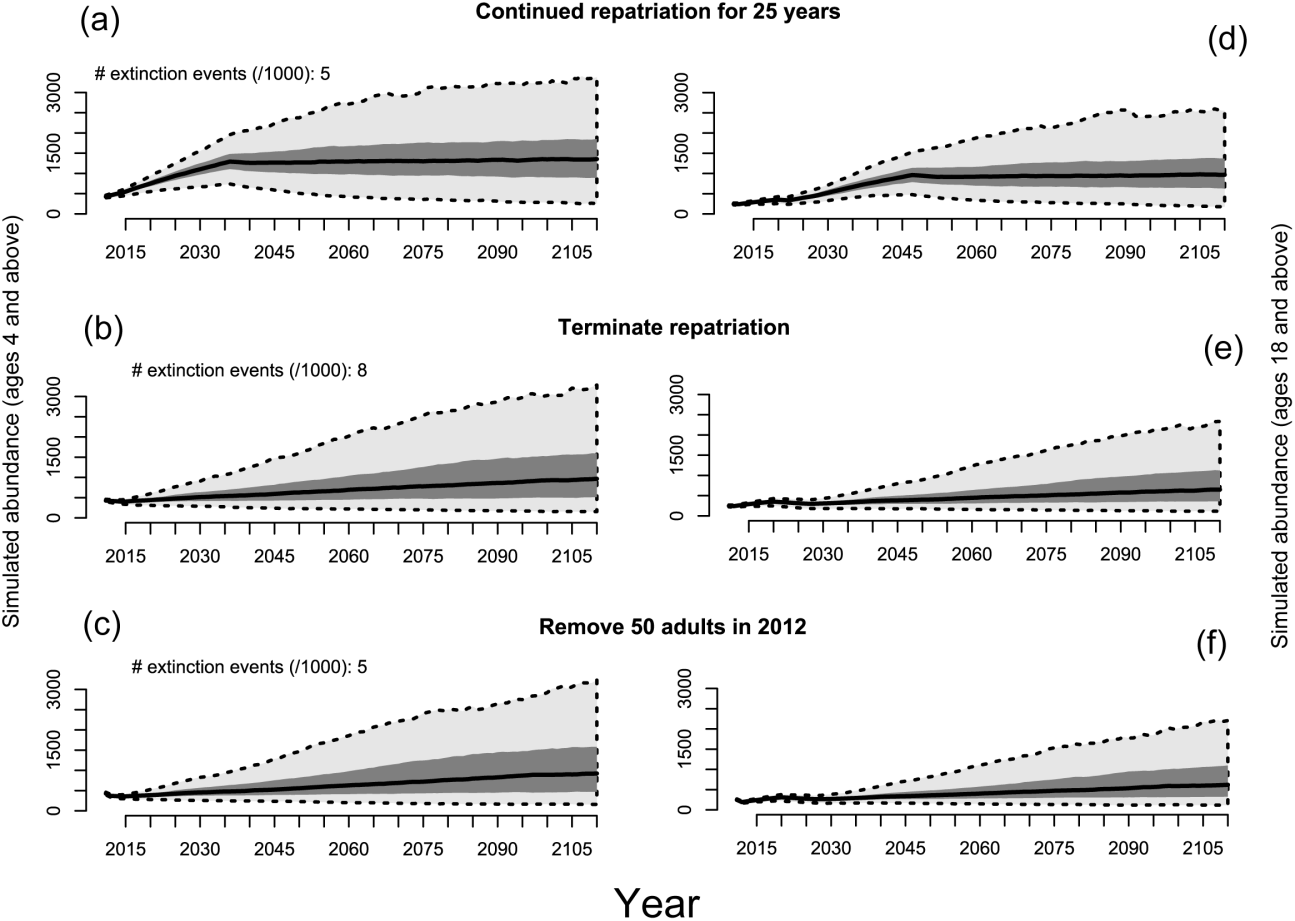
Summary of projected 100-year population dynamics for the repatriated giant tortoise population on Española Island, Galapagos, for (A, B, C) all female tortoises ≥4 years old, and (D, E, F) only adult female tortoises ≥18 years. Vital rates for the projection models were developed largely from a 32-year-capture-recapture database and carrying capacity (K) was determined using tortoise densities measured at field plots and modeled as a function of vegetation characteristics and distance from release sites. We modeled three management scenarios: (A, D) continued release of captive-raised tortoises for 25 years, (C, E) termination of tortoise repatriation, and (C, F) removal of 50 reproductive adult females at the beginning of the simulation (e.g., to accelerate repatriation efforts on other islands).

Population projections built from vital rates estimated from the mark-recapture data suggest that the benefits of continuing tortoise repatriation efforts to Española in terms of population viability would be very low. Continued repatriation for 25 more years, termination of repatriation, and termination of repatriation coupled with one-time removal of 50 adults (for possible translocation to other islands) yielded nearly equal and negligible extinction risk estimates over the 100-year time frame (Figs. 7a–c). In terms of restoring the role of giant tortoises as ecosystem engineers, however, the scenarios’ outcomes contrasted in a substantive manner: carrying capacity was projected to be reached under the continued repatriation scenario by 2075 versus by 2100 for repatriation termination and by 2115 for removal of 50 adults in 2012 with termination of repatriation. This pattern was even more pronounced when adult (female) abundance was examined in isolation: adult abundance plateaus at ca. 1000 adult female tortoises (ca. 2000 total adults) by the year 2045 under the continued repatriation scenario (Fig. 7d), whereas adult abundance only reached half this level by the end of the simulation period under scenarios in which repatriation is terminated (Fig. 7e, f).

## Discussion

The science of species reintroduction has typically focused on the initial steps of a reintroduction project: how to physically release the animals [41], how to improve genetic mixing of the small initial population [42], how suitable receiving habitat is for a release [43], and how to prevent excessive dispersal away from the intended release site [44]–[45]. Although these considerations are important for initial re-establishment of any population, how to generate wide-scale re-delivery of ecosystem-level processes via the introduced species is more complicated and rarely examined. In the case of the giant tortoise repatriation on Española Island in Galapagos, we determined that the re-introduced tortoise population was likely now self-sustaining over the long-term, but realization of ecological goals for the tortoise re-introduction program (notably, full occupation of the island by tortoises and the restoration of the island’s once-dominant herbaceous and graminoid vegetation) will require considerably more time and perhaps further intervention in the form of habitat management.

More specifically, our analysis indicated that the repatriated Española tortoise population is secure from a strictly demographic perspective: extinction risk was very low (<1%) with or without continued repatriation, and population growth rates were weakly positive. We are confident in our population reconstruction in part because the estimate of tortoise population size derived from plot-based counts of tortoises yielded an estimate of population size that corresponded to the final abundance estimate derived in a completely independent manner from analysis of capture-recapture data. This analysis was made possible by a rigorous monitoring program conducted in parallel with and since the inception of the reintroduction effort 32 years ago, and because ancillary sources of information were available (e.g., plot-based analyses of vegetation characteristics and tortoise densities) from which we could estimate carrying capacity.

Our demographic analyses for the Española population imply that approximately half of tortoises released on the island since 1975 were still alive in 2007, and that estimated fecundity rates were generally sufficient to offset mortality, resulting in modestly positive population growth even if the repatriation program were to be terminated (Fig. 6). Similar, high intrinsic population growth rates were reported for Aldabran giant tortoises, whose population rebounded from very low numbers in the early 20th century (when they were in the hundreds or low thousands [46]) to over 100,000 tortoises by the 1970s after cessation of severe harvesting pressures, which implies a discrete annual population growth of at least 1.07 [46]–[47]. The Aldabra example and our case study of Española tortoises together illustrate that giant tortoise conservation efforts starting from very few individuals have the potential for relatively rapid success if the deterministic factors that caused population decline originally are ameliorated.

The apparent decline in the survival rate of release cohorts we observed since ca. 1994 (Fig. 6a), along with decreasing growth rates and body condition, may be evidence, however, that resources on Española island are becoming limiting to continued population growth. In other words, the population may now be at or near carrying capacity of the area occupied. Two caveats may apply to this conclusion. First, declines in apparent survival could be due to emigration of young tortoises from the core area that is the focus on tortoise monitoring, perhaps motivated by density-dependent food competition among tortoises in a growing population. This said, recent surveys outside the tortoise zone [48] have revealed very little sign (droppings, bedding sites, trails) of tortoises elsewhere on the island. Second, the reduction we observed in growth rates and condition over time could be simple artifacts of ontogenetic changes in carapace length in an aging population given that this is a prominently saddle-backed species whose distinct morphology is manifested after sexual maturity [49]. However, the magnitude of relative change of carapace length (the measure of size used in this study) versus other measures of body size is indistinguishable in male and very modest in female saddle-backed tortoises between younger and older individuals [50]).

Population growth is generally not limited by environmental constraints during early phases of reintroduction efforts because densities are below carrying capacity [51]–[52], with population limiting factors not manifesting their effects until population densities increase. With high estimated adult, subadult and juvenile annual survival rates of ca. 98% throughout the reintroduction effort on Española Island, and in the absence of opportunity for migration (as seems to be the case for this island population where suitable habitat is limited to a small sector of the island), low survival of hatchlings and very young individuals is likely to limit population growth as the population approaches carrying capacity. This was evidenced by the generally low estimated survival (≤50%) of tortoises released since 1994, after which population growth rates tended to be reduced (Fig. 6a). While juvenile survival rates estimated for giant tortoises in this study are not atypical for turtles and tortoise populations experiencing stable growth [40], higher juvenile survival rates are likely required to boost population growth rates [53].

We estimated a low probability of extinction for the Española tortoise population under all of the future management scenarios we assessed. We have used an admittedly short time-frame (100 years) for considering extinction risk for such a long-lived animal (generation time is estimated at ca. 50 to 60 years on the basis of our vital rate estimates) yet modeling time frames beyond 100 years are of little relevance to managers and conservation planners [54]. Clearly, projecting the success of reintroduction efforts can be difficult for long-lived species such as tortoises, whose reproduction can be delayed for a decade or more and for which high adult annual survival often obscures critical population dynamics [53]. For example, few reintroduction projects for long-lived species like tortoises and turtles have monitored populations long enough to estimate adult survival (with some exceptions [41]), let alone to estimate carrying capacity and to project density-dependent declines in population growth rates, yet such estimates are critical for confirming reintroduction success and estimating the long-term ecological effects of reintroduction efforts.

Despite evident achievement of population viability through this repatriation program, significant constraints evidently remain on the tortoise population’s ability to achieve high densities and extensive spatial distribution. More specifically, the tortoises’ preferred habitat, areas with high adult cactus density and low woody plant density, is currently limited across the island, and consequently the tortoises’ movements are restricted to a small portion of the island despite an intrinsic movement capacity to expand to a far larger area than the species currently occupies. Our plot-based analysis of tortoise-cactus-woody-plant interactions supports the general hypothesis that woody plants have negative impacts on tortoises. This likely occurs because woody plants block tortoise movement thereby increasing travel costs and restricting access to habitat. Woody plants also reduce cactus recruitment, likely through light and water competition and perhaps by mediating tortoise movement thereby limiting dispersal and germination of cactus seed [16].

The current predominance of woody plants appears to be anomalous in the context of the history of vegetation on the island given that temporal trends in stable carbon isotopes of soils indicated a pronounced shift toward woody plants in the recent history of Española Island’s plant community. This inference relied on the clear separation we observed in δ^13^C values for woody and herbaceous plants but also relies on a reasonable but impossible-to-validate assumption that the plant species sampled in the present also were those that dominated in the past. The cause of the recent shift towards woody plants cannot be determined from data available to us because in addition to the herbivory by goats and centuries-long effective absence of tortoises, climate change may have altered plant communities and the δ^13^C values expressed in the soil profile. This said, other studies in the Galapagos have indicated a change in the historical plant community composition with the reduction in giant tortoise populations and invasion of exotic herbivores [55]. Moreover in other semi-arid ecosystems, including ones that share some of the woody plant genera that dominate Española Island, the loss of a native herbivore and the introduction of an invasive herbivore has resulted in woody plant encroachment to an extent that the ecosystem cannot evidently recover without intervention [28]. Given the current widespread dominance of woody plants on Española Island, it is possible that the system has already reached a new, stable and woody-plant-dominated state that may require extensive woody plant removal or other habitat management to transition to a more mixed woody plant-cactus-grassland state [56].

The status of cactus on the island also remains of particular concern, not just because the species itself is of conservation concern due to its highly restricted distribution and reduced abundance due to historical goat impacts, but also because tortoises are heavily dependent on it. There are currently only an estimated 1310 adult cacti in the zone occupied by tortoises on Española Island, or just 1–2 cactus per tortoise. Moreover, our density estimates for cactus on Española Island of 1.7 juvenile cactus/ha, 0.4 subadult cactus/ha, and 1.0 adult cactus/ha (3.1 total stems/ha) are anomalously low for Galapagos in comparison to, for example, those on other comparable arid islands (Pinzón and Santa Fe Islands), which have stem densities of about 300–440/ha, that is, two orders of magnitude higher [57]. Our surveys also revealed a stage structure among cacti that is dramatically skewed to adults, thereby indicating a constraint on recruitment of young cacti into the adult population. Cactus populations elsewhere in Galapagos show a typical juvenile-biased size distribution [58], with a clear dominance of younger age classes typical of non-declining plant populations.

The tortoise re-introduction does appear to have a net positive impact on the cactus population, itself of conservation concern due to destruction by goats now removed. In this study we observed that the cactus population is increasing in one of the primary tortoise release sites, Las Tunas, confirming the suggestion [16] that tortoises have a modest yet positive impact on cactus populations. However, the population growth rate of adult cacti observed (about 7% over the last decade) is considerably less than the per decade rate at which the tortoise population has grown over the last four decades (about 200 tortoises or 33% population growth per decade). Notably tortoise population growth is comparable to the growth rate of subadult and juvenile cacti observed herein, or 37%. Only adult cactus substantially produce resources needed by tortoises (fruits, pads, shade) and the disparity of population growth rates of tortoises versus adult cacti indicate that ratios of tortoises to adult cacti will increase with time; however, as long as adult cactus survival remains high we predict a slowly improving status for the cactus population resulting from interactions with reintroduced tortoises, which foster recruitment of young cacti that will eventually transition to adult status producing resources (pads, fruits and shade) of use to tortoises.

Given that tortoises are now restricted to a small portion of Española Island and that tortoise habitat has likely degraded, it comes as little surprise that carrying capacity estimated for tortoises on Española appears to be quite low: approximately 2100 tortoises for the 1250 ha “tortoise zone” or 1.7 tortoises/hectare. This is striking given that tortoise densities on other islands in the archipelago have been reported as high as 8 tortoises/hectare [59]. Therefore, continued reintroductions of juvenile tortoises to the island would seem ill-advised. Currently juveniles have low survival rates and are therefore recruited infrequently into the population and likely play only a small role in sustaining it.

Instead of further repatriations, a more useful approach to improve the long-term outlook for this species to expand its ecological role on the island would be to improve habitat in such a way that the tortoises, now with significant numbers of older repatriates evidently reproducing, can colonize more of the island. More specifically, woody plant density is evidently a limiting factor to population growth given that a 50% reduction in woody plant density was projected to correspond to a 52% increase in the tortoise carrying capacity. Achieving this scale of restoration via manual removal of woody plants (the only currently available option) on half the island would, however, be extraordinarily expensive and logistically difficult given the remoteness of the island. Prescribed burns are not currently used as a habitat management tool in Galapagos given the unclear role of natural fires as an historical agent of natural disturbance in the archipelago [60], thus mechanical or chemical means may be required to reduce woody plant extent to levels more consistent with the recent history of the plant community on the island (Fig. 3).

Given the depleted and evidently declining status of tree cactus on the island combined with its importance to tortoises, a special focus on cactus management may be warranted given the near absence of cactus on much of the uncolonized parts of the island. Large-scale reseeding of cactus, now feasible given the availability of new technologies (e.g., [61]), could increase cactus recruitment, growth and survival dramatically. Given light competition between cactus and woody plants, cactus out-planting would best coincide with woody plant control (see Fig. 2c for potential cactus restoration areas with low slopes and low woody plant cover). Because tortoises are suspected to not only disperse cactus seeds and thereby increase recruitment but also mechanically flatten small cactus and thereby reduce cactus recruitment [19], a phased approach involving clearing of woody plants in new zones of the island unoccupied by tortoises and distant from occupied areas coupled with cactus out-plantings would be most effective. Timing of tortoise colonization could be controlled by establishing corridors to cactus groves in areas cleared of woody plants once sufficient number of out-planted cactus had grown to “tortoise-proof” size (subadult and older stages), that is, a decade or more after planting [62].

The reintroduction of an ecosystem engineer can have indirect benefits for other species of conservation concern [6],[8]. We have demonstrated that in the case of the Española tortoise in addition to recruitment benefits conferred to the endangered Opuntia cactus that is a primary tortoise food source, woody plant reduction could also aid the Waved Albatross (*Phoebastria irrorata*), a critically endangered seabird that effectively only nests on Española Island and that is likely limited by access to open-ground take-off and landing sites [48],[63]. Tortoises may be able to reduce or at least control woody plant density near albatross sites, but the current tortoise zone only marginally overlaps with that of the Waved Albatross, which are restricted to the southern part of the island [64]. Española tortoises and Waved Albatross may have a long co-evolutionary relationship: the absence of actual nest-building by albatross and their habit of rolling their eggs may be a response to the threat of trampling by tortoises [65]. The potential cross-species benefits of this tortoise reintroduction supports the more general claim that re-establishment of ecosystem engineers should be prioritized in conservation and ecological restoration [66]–[67]. For these benefits to be realized in the case of Española Island, however, we conclude that reinstatement of Española giant tortoises as fully functioning ecosystem engineers and not simply a viable population will likely require large-scale habitat restoration efforts in concert with further population restoration.

## Supporting Information

Appendix S1 **Contains a detailed description of demographic model and population projection model used.**
(DOCX)

Appendix S2 **Contains R and WinBUGS code for estimating survival from 32 years of tortoise capture recapture data gathered on Española island.**
(DOCX)

Appendix S3 **Contains R code for projecting population dynamics for the** ***in situ*** **population of Española giant tortoises.**
(DOCX)
