# Correction: Demographic Outcomes and Ecosystem Implications of Giant Tortoise Reintroduction to Española Island, Galapagos

**DOI:** 10.1371/journal.pone.0114048

**Published:** 2014-11-14

**Authors:** The PLOS ONE Staff

Figures 2, 3, 6 and 7 are incorrect. The authors have provided a corrected version of each figure here.

**Figure 2:**
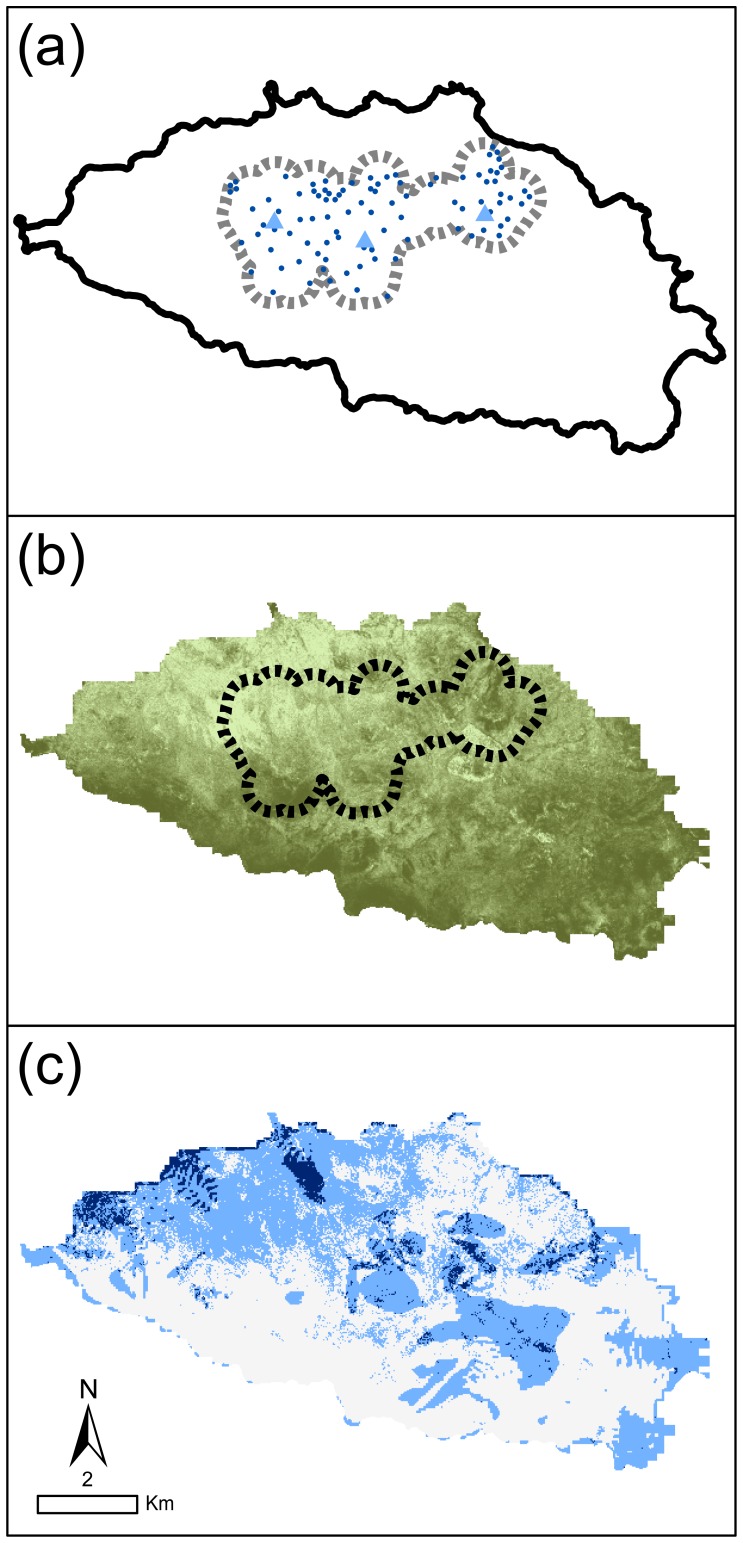
Study areas on Española Island. (A): gray dashed line is “tortoise zone” or maximum extent of tortoises observed on the island since the repatriation program was initiated; black circles are 2010 survey plot locations for tortoises, cactus, and woody plants; gray triangles are tortoise introduction sites (West: Tunas, central: Cacos, East: Gardner). Woody plant cover on Española Island (B; darker shades indicate higher percent woody plant cover), classified using cloud-free Quickbird satellite imagery (December 29, 2006, 0.6 meters resolution) into areas with and without woody vegetation using a supervised classification calibrated with plot woody plant density estimates (IDRISI Andes 5.0) (percent cover is the percent vegetated cells per 24m x 24m area). Potentially highly suitable cactus restoration areas (C) with low slopes (<2.06 degrees, bottom quartile of available slopes) and low woody plant cover (<52% cover woody plants, bottom quartile of available woody plant cover); black areas have both low slopes and low woody cover, gray areas have either low slope or low woody cover.

**Figure 3:**
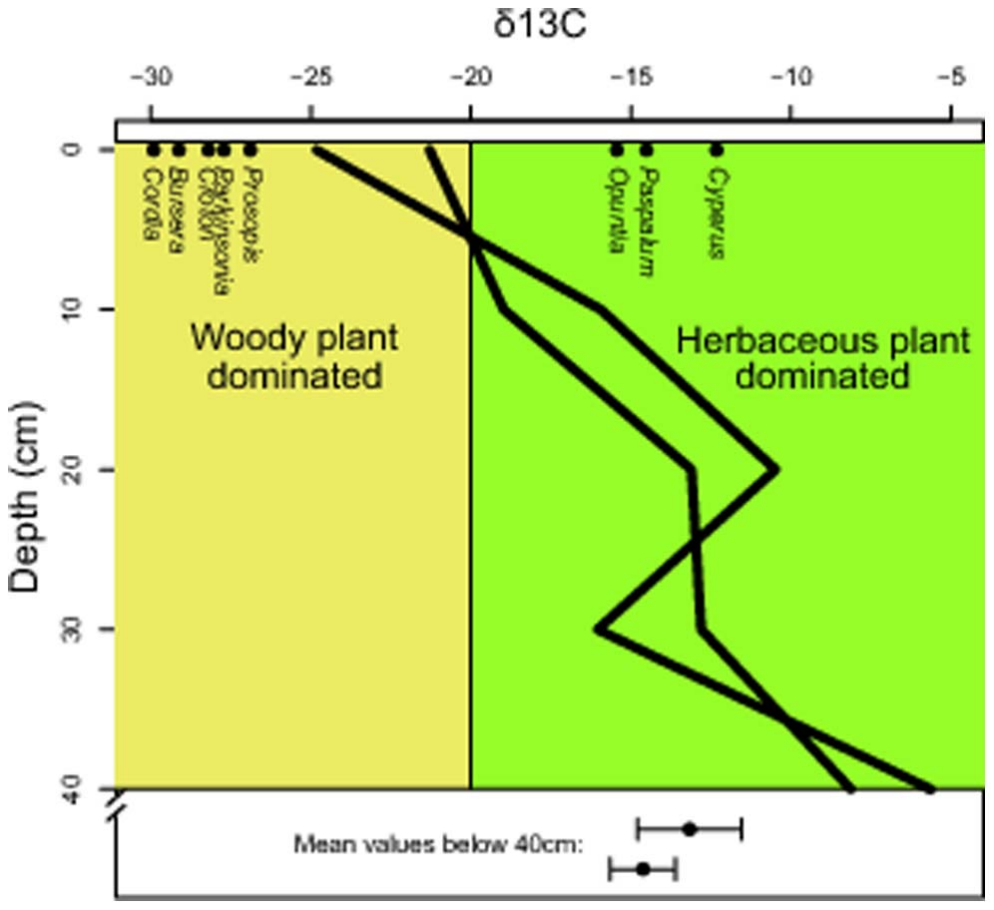
Carbon isotope signatures (δ13C values) of soils at increasingly greater depths in two soil pits (Gardner and El Caco) on Española Island, Galapagos. Radiocarbon dating indicated that depths of 40 cm are approximately 500 years BP; therefore, samples below the 40 cm depth are grouped to show pre-disturbance averages. Points are δ13C values of the island’s dominant woody plants (*Bursera, Cordia, Croton, Parkinsonia, Prosopis*) and herbaceous plants (*Cyperus* and *Paspalum*) as well as cactus (*Opuntia*) (averaged across 10 samples of each, with standard errors for all means too miniscule to be evident in this graphic).

**Figure 6:**
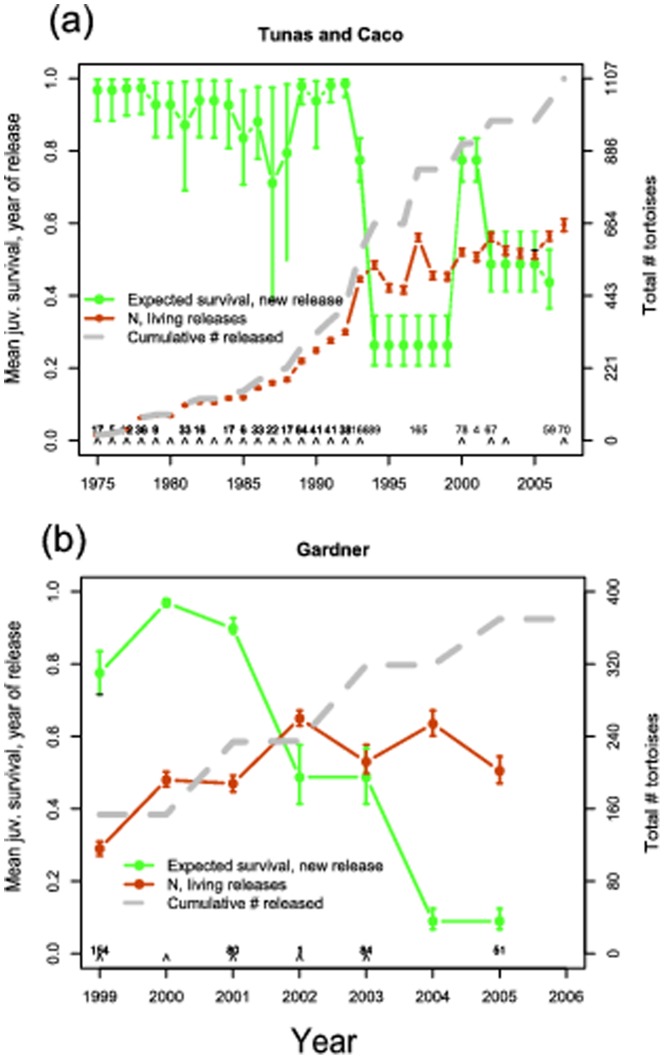
Estimates derived from a 32-year capture-recapture data set of abundance and vital rates of giant tortoises reintroduced to Española Island, Galapagos depicted by release sites (see Fig. 1): Tunas and Caco combined (A) and Gardner (B). Closed circles depict expected survival rates for newly released juveniles, the dashed line cumulative number of tortoises released, and open circles the estimated number of released individuals remaining. Release years are denoted with carets located above the x axis, and the numbers above the carets indicate the total number of tortoises released during each repatriation event.

**Figure 7:**
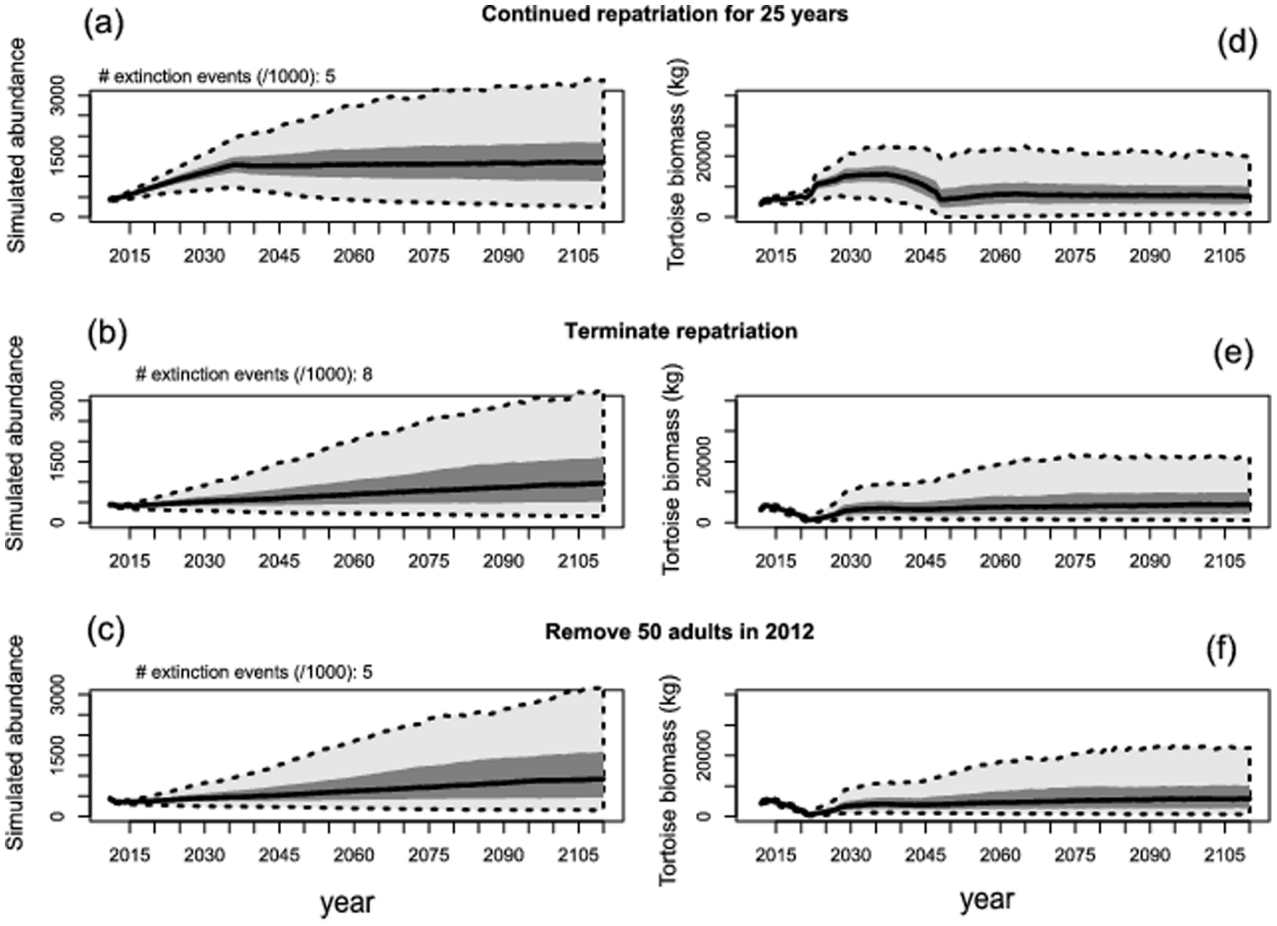
Summary of projected 100-year population dynamics for the repatriated giant tortoise population on Española Island, Galapagos, for (A, B, C) all female tortoises ≥4 years old, and (D, E, F) only adult female tortoises ≥18 years. Vital rates for the projection models were developed largely from a 32-year-capture-recapture database and carrying capacity (K) was determined using tortoise densities measured at field plots and modeled as a function of vegetation characteristics and distance from release sites. We modeled three management scenarios: (A, D) continued release of captive-raised tortoises for 25 years, (C, E) termination of tortoise repatriation, and (C, F) removal of 50 reproductive adult females at the beginning of the simulation (e.g., to accelerate repatriation efforts on other islands).
