# Supplementary material for: Demographic Outcomes and Ecosystem Implications of Giant Tortoise Reintroduction to Española Island, Galapagos
Source: PLoS One. 2014 Oct 28;9(10):e110742. doi: 10.1371/journal.pone.0110742 (PMC4211691; doi:10.1371/journal.pone.0110742)
Supplement: Appendix S1 — Contains a detailed description of demographic model and population projection model used. (DOCX) [file pone.0110742.s001.docx]

Gibbs, J.P. et al. Giant Tortoise Reintroduction to Española Island, Galápagos: Demographic Outcomes and Ecosystem Implications.

# Appendix S1: Detailed description of demographic model and population projection model

## Capture-recapture analysis

Since repatriation of Galápagos giant tortoises began on the island of Española in 1975, researchers from Parqué Nacional Galápagos (PNG) performed regular capture-recapture surveys to evaluate repatriation success. From 1975 to 1998, surveys and releases were confined to the northwestern region of Española (associated with release sites “Tunas” and “Caco”). In 1999, a third release site (“Gardner”) was added in the northeastern region of the island. The result was a 32-year capture history dataset comprising 570 recaptured individuals associated with the “Tunas and Caco” region (1975 to 2007), and an 8-year capture-history dataset comprising 309 individuals associated with the “Gardner” region (years 1999 to 2007). Tortoise surveys were conducted each year of the study period except 1994 to 1999, 2001, and 2004-2006, resulting in 22 years of recapture information, during which an average of 84 tortoises was captured per survey year (range: 7 to 227, generally increasing with time due to increasing tortoise abundance). Individuals were captured on average 2.2 times each (median = 1, minimum = 1, maximum = 21).

In addition to this extensive mark-recapture dataset, the total number of individuals released each year at the three release sites ("Tunas”, “Caco”, and "Gardner") was known. Many releases that were never recaptured were not included in the capture-recapture dataset. Releases of juvenile tortoises occurred during each of 20 years within the study period (1975 to 2007), and comprised from 12 to 168 individuals that varied from 2 to 7 years in age. We merged information about the numbers of tortoises released each year with the capture history data set, producing a comprehensive capture history data set covering the history of the repatriation program on Española island. Since the year and age of release was known for all individuals in the mark-recapture dataset, it was a relatively simple task to match individuals in the capture history with appropriate individuals in each release cohort on the basis of the recorded age-at-release of each individual in the capture recapture dataset (the release year was known for all individuals in the capture recapture dataset). All individuals in the release cohort that were not represented in the capture recapture data set were assumed to have never been recaptured, and were assigned zeros for all subsequent sampling bouts in the merged capture history dataset.

During the first 17 years of the repatriation program (1975-1991), tortoises were assigned unique marks using a combination of notches (made with machete or metal file) carved onto the tortoises’ marginal scutes. Beginning in the year 1992, a duplicate marking system was introduced consisting of numbers branded on the rear costal scutes combined with insertion of passive integrated transponder (PIT) tags (AVID Identification Systems Inc., Norco, California, USA) into the left rear leg of each tortoise. However, due to the change in marking system and a failure to systematically link the previous marking system with the PIT tagging sytem, it was not possible to link individuals in the pre-1992 period directly to corresponding individuals in the post-1991 period. For those individuals that were released prior to 1992 but captured after 1991, and therefore appearing in both the 1975-1991 and the 1992-2007 survey periods, we developed a system for merging the capture histories from these two time periods using information about the age of individuals in the two capture history sets. First, we inferred the approximate age of each individual in the post-1991 dataset on the basis of the recorded age at first capture (estimated on the basis of an annulus count). Because age could not be estimated exactly (there was some associated error in counting annuli), we reclassified all individuals into coarser age classes: [0-4), [4-7), [7-10), [10-15), and [15-30]. We then linked individuals in the post-1991 data set with individuals in the pre-PIT data set such that they matched with individuals of approximately the same age during the transition year (1991-1992).

A problem with this system is that some individuals in the pre-PIT data set likely died prior to 1992, and therefore could not be recaptured in the post-PIT data set. Therefore, when "breaking ties" (i.e., when there were multiple individuals in the pre-PIT dataset matching the inferred 1992 age class), we assumed individuals captured more recently in the pre-PIT dataset were more probable matches for individuals in the post-1991 data set than individuals that had not been captured for many years. Because this process of merging datasets will influence survival estimates for the pre-PIT period (e.g., if individuals in the pre-PIT dataset that had not been captured for many years were matched preferentially with individuals in the post-1991 dataset, survival rates the pre-PIT dataset would be artificially inflated), we chose to develop a system that (conservatively) deflated survival rates in the pre-PIT period (i.e., maximized mortality rates). To accomplish this, we formulated our merging system such that individuals captured more recently in the pre-PIT dataset were given more preference as a match than would be expected on the basis of preliminary survival estimates for each individual (determined on the basis of CJS models for the pre-PIT dataset). Although imperfect, this system allowed us to develop a merged data set encompassing all years of the tortoise repatriation project on Espanola without introducing any important biases in survival estimation. In fact, by merging information about known releases with the observed capture recapture dataset (which excluded many releases that were never recaptured) we eliminated an important potential source of bias in survival estimates for juvenile tortoises (that is, if all tortoises that were recaptured were included in the data set but some tortoises that were never recaptured were excluded, then estimates of juvenile survival would be biased high).

### Analytical approach

We estimated survival rates from the merged capture history data set for 'Tunas and Caco' and 'Gardner' using the Bayesian state-based framework described by Royle and Dorazio (2008), analogous to a standard Cormack-Jolly-Seber model. We estimated survival (φ) separately for juveniles (<8 y.o.) and subadults/adults (≥8 y.o.). Survival rates were estimated separately for each 4-year time period (subadults/adults) and 2-year time periods (juveniles), allowing for temporal variation in survival (environmental stochasticity and potential trends in survival rates). Survival at year-of-release was estimated separately for each release year. Because our Bayesian modeling framework explicitly tracked the status of each individual released tortoise (i.e., alive or dead) each year, we were able to compute the annual abundance of released tortoises (excluding native-born individuals) at both major release areas (“Tunas and Caco” and “Gardner”) as the sum of living individuals occupying each of these release areas.

Parameter estimation was performed by Markov-Chain Monte Carlo (MCMC) using WinBUGS 1.4 (Lunn et al. 2000; <http://www.mrc-bsu.cam.ac.uk/bugs/>). WinBUGS models were accessed, processed and called from the R environment using the R2WinBUGS package for R (Sturtz et al. 2005; R Development Core Team 2012). Uninformative uniform (0,1) prior probability distributions were assigned to all parameters representing probabilities and uniform (0.01,5) priors were assigned for (hyper)parameters representing standard deviations for logit-normal random effects. We performed 15,000 MCMC iterations, discarding the initial 5,000 samples as a burn-in to give the algorithm time to converge on the joint posterior distribution, and saving every 10^th^ iteration to reduce serial autocorrelation among samples (McCarthy 2007, Appendix C therein; Bolker 2008, Chapter 7 therein). We summarized posterior distributions for all parameters using the empirical mean of all MCMC samples as point estimates and the 2.5 and 97.5 percentiles of the MCMC samples as a “credible” interval (McCarthy 2007) (Figure S1.1). Our R and WinBUGS code and a detailed description of the statistical model are in Appendix S2.


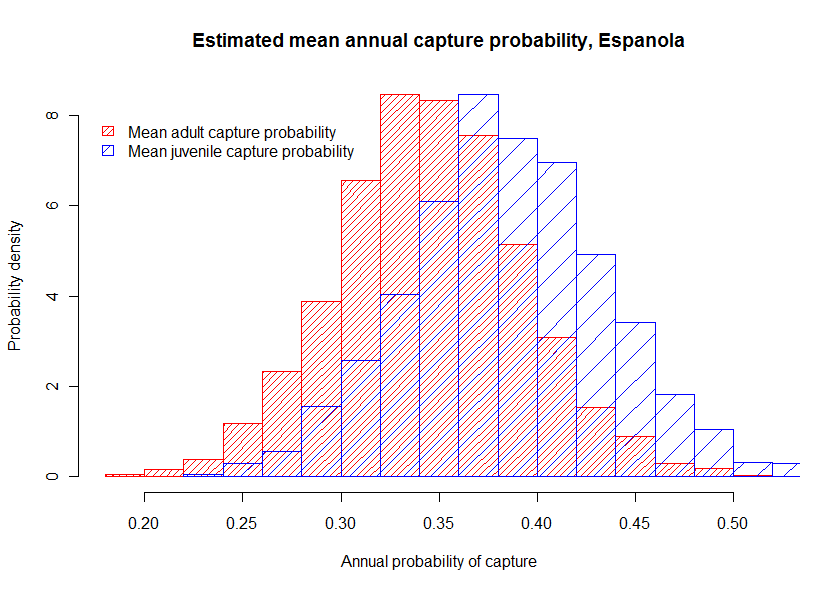


*Fig. S1-1. Mean annual capture probability for giant tortoises on Espanola island during repatriation efforts from 1975-2007. Parameter estimates were based on a long-term (32-year) capture-recapture database spanning the repatriation efforts on Española. All parameter estimates were derived from a Bayesian Cormack-Jolly-Seber model*

## Population projection model

Galápagos giant tortoises have been released regularly on Española since 1975 in an effort to repatriate the island. With over 1000 captive-raised tortoises released over the past 35 years, one of our primary objectives was to evaluate the marginal benefits of continued repatriation efforts at Española. To evaluate the consequences of termination vs. continuation of repatriation, we built a simple model of giant tortoise population dynamics, with survival parameters sampled directly from the output of the capture-recapture analyses described above. Population projection scenarios included continuation vs. termination of the repatriation program, and translocating 50 adult tortoises from Española to others islands to reestablish giant tortoises elsewhere where they have gone extinct (both management options under consideration).

We projected potential future tortoise population size by incorporating survival estimates from the mark-recapture study into a matrix-based projection model of giant tortoise population dynamics. Survival rates of tortoises aged 1-2 were not estimable from our capture history database and were assigned a range of values typical of very long-lived turtle species (see Pike et al. 2008). To account for demographic stochasticity, annual survival for each age class was modeled as a Binomial trial with N computed as the number of tortoises in the previous age class in the previous year and p = the appropriate stage-specific mean survival rate. Aside from survival rates, critical input parameters for population-projection modeling include total fertility (an amalgam of clutch size, clutch frequency, egg viability, and hatchling survival rate), initial abundance, and carrying capacity (K). Initial (2011) abundance for Española projection models was approximated using the estimated total number of released individuals that were alive in 2007 (derived from capture-recapture analyses, which ignored native-born individuals). Carrying capacity (K) for Española was determined on the basis of a regression model of tortoise densities observed at survey plots in 2010 (see text).

We used a modified "ceiling" density dependence model, which assumed no compensatory increases in population vital rates below carrying capacity (note that as a reintroduced population that was likely at or below carrying capacity for much of the study period, observed population growth rates may in fact represent an value intermediate between equilibrium growth [R=1], and the intrinsic maximum rate of growth, R_max_). In our implementation of a ceiling model, abundance above carrying capacity resulted in reduced survival of young juveniles and reduced fecundity. The result was a "soft" ceiling to population growth above carrying capacity, such that abundance could exceed carrying capacity for short periods.

The proportion of native-born individuals on Española that are female is largely unknown, as sex is determined by the nest-site temperature regime (which has not been evaluated). Therefore the proportion of females in the population was assigned a wide range from 0.3 to 0.7, in which a single value was sampled for each simulation run. Total fertility rate (total number of yearlings produced per reproductive adult) was computed as the product of four parameters: mean proportion of females in the population, mean number of eggs produced per female, mean hatching success rate, and mean neonate (hatchling) survival. All parameter values used in our population models, and the information sources for these parameter values, are presented in Table 1 in the main text. Mean # eggs produced per female per year was sampled independently from 3 to 10 for each simulation run (*n* = 100), on the basis of the observed numbers of eggs produced by female Española tortoises in the captive breeding program (Márquez et al. 1999). The remaining fertility parameters were estimated using an approximate-likelihood technique (described below).

Although we were not able to use the capture-recapture data to estimate unknown fertility parameters directly (i.e., hatching success and neonate survivorship), we devised an approximate-likelihood technique for estimating fertility rates from the fraction of native-born tortoises observed each year (recorded from capture-recapture surveys). To do this, we simulated population dynamics on Española given the known history of releases (year of release, number released, and age at release) from 1975 to 2010, using survival rates derived from the capture-recapture analyses. We ran 100 simulations for each of ten scenarios in which the product of egg viability and hatchling survival was assigned values ranging from 0.0064 (both parameters set at 0.08) to 0.16 (both parameters set at 0.4). Mean cumulative numbers of “nativos” (native-born individuals; on the basis of 100 simulation runs) were plotted alongside “observed” native-born recruits (calculated as the product of population size and the observed proportion of “nativos” observed during capture-recapture surveys), and a plausible range of fertility parameter values was chosen based on the visual concordance of simulated vs. observed numbers of “nativos” (see Fig. S1-2). From this approximate-likelihood analysis, we concluded that the product of egg viability and hatchling survival at Espanola was between 0.023 (equivalent to both parameters set at 0.15) and 0.066 (equivalent to both parameters set at 0.26).

Recruitment was modeled as a Poisson process (without specific evidence to suggest overdispersion or underdispersion, we chose the simplest and most widely-used distribution for discrete, positive random variables; see Bolker 2008), with mean annual numbers of yearling recruits calculated as the product of previous-year adult abundance and (site-specific) per-capita fertility rates. Lacking information on the covariance structure of the four components of fertility, we conservatively assumed that all four parameters were perfectly correlated (e.g., simulation runs in which mean egg production was very low also had very low hatching success, etc.), which is likely to underestimate population stability.


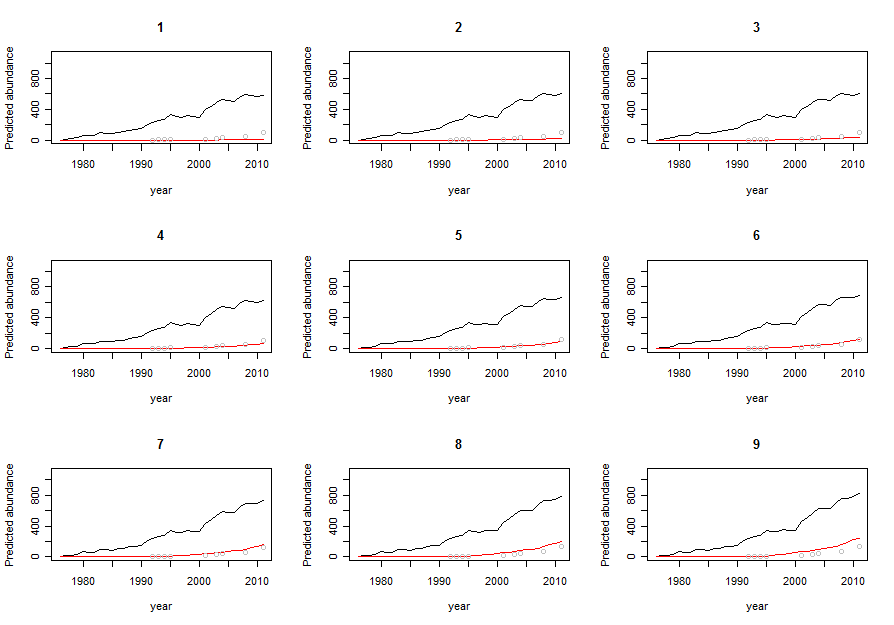


*Fig. S1-2: Approximate likelihood estimate of per-capita fecundity. Nine plausible fecundity scenarios, varying from low to high. Scenarios 4 through 7 match best with observed fraction of presumed nativos recorded in each sample, corresponding to fertility parameters (product of egg viability and hatchling survival at Espanola) between 0.023 (equivalent to both parameters set at 0.15) and 0.066 (equivalent to both parameters set at 0.26). Open circles represent expected number of nativos based on observed fraction captured in field samples, while the red line illustrates the expected fraction nativos under each simulated fecundity scenario.*

To estimate population stability under continuation vs. termination of the repatriation program on Española, we ran 100 separate population simulations for 150 years each. Simulated populations were considered functionally extirpated after reaching a quasi-extinction threshold of 75 adults or fewer at any point during the simulation, although we allowed all simulations to proceed for the full 150-year duration. For the continued-repatriation scenario, we simulated the release of 50 captive-raised 5-year-old individuals each year for 25 years (from 2011 through 2036). We estimated extirpation probability as the proportion of simulation runs reaching the quasi-extinction threshold after 150 years. All population projection models were coded in the R statistical computing language. R scripts for performing this analysis is presented in Appendix S3.

## Literature Cited

Bolker, B.M., 2008. Ecological models and data in R, Princeton University Press, Princeton, NJ.

Márquez C., Cayot L.J. & Rea S. 1999. La Crianza de tortugas gigantes en cautiverio: Un manual operativo. A & B Editores, Quito, Ecuador.

McCarthy, M. A. (2007). Bayesian methods for ecology. Cambridge University Press.

Pike, D.A., Pizzatto, L., Pike, B.A., Shine, R., 2008. Estimating survival rates of uncatchable animals: the myth of high juvenile mortality in reptiles. Ecology 89, 607-611.

R Development Core Team. 2012. R: A language and environment for statistical computing. R Foundation for Statistical Computing, Vienna, Austria. ISBN 3-900051-07-0, URL http://www.R-project.org/.

Royle, J.A., and Dorazio, R.M., 2008. Hierarchical modeling and inference in ecology. Academic Press, Amsterdam, the Netherlands.

Sturtz, S., Ligges, U., and Gelman, A. (2005). R2WinBUGS: A Package for Running WinBUGS from R. Journal of Statistical Software, 12(3), 1-16.
