# Supplementary material for: Demographic Outcomes and Ecosystem Implications of Giant Tortoise Reintroduction to Española Island, Galapagos
Source: PLoS One. 2014 Oct 28;9(10):e110742. doi: 10.1371/journal.pone.0110742 (PMC4211691; doi:10.1371/journal.pone.0110742)
Supplement: Appendix S2 — Contains R and WinBUGS code for estimating survival from 32 years of tortoise capture recapture data gathered on Española island. (DOCX) [file pone.0110742.s002.docx]

Gibbs, J.P. et al. Giant Tortoise Reintroduction to Española Island, Galápagos: Demographic Outcomes and Ecosystem Implications.

# Appendix S2: R and WinBUGS code for estimating survival from 32 years of tortoise capture recapture data at Española island

## R code

#################

#Espanola mark recapture analysis, R script

#################

######################################

##### Set data directory

setwd("[[your directory]]")

#######################################

###### load dependencies

library(R2WinBUGS)

library(Hmisc)

library(mra)

library(msm)

#######################################

####### Read in data

#### CAPTURE HISTORY

caphist=read.csv("tortcr.csv",header=F)

caphist=caphist[1:570,1:23]

caphist=as.matrix(caphist) ### capture history: includes all recaptured individuals but not all releases

nan=nrow(caphist) # rows of caphist

ns=ncol(caphist) # columns of caphist

yrs.since <- c(1,1,1,1,1,1,1,1,1,1,1,1,1,1,1,1,1,1,2,6,2,1,4) # years separating each survey bout in the main capture history dataset

#### SEX

female=as.matrix(read.csv("tortcr_female.csv",header=F)) ### matrix of sex (from James' analysis in mra)

male=as.matrix(read.csv("tortcr_male.csv",header=F))

### AGE AT RELEASE

agerel=as.matrix(read.csv("tortcr_ageatrelease.csv",header=F)[1:nan,1:ns]) ## matrix of age at release, from James' analysis in mra

agerel=as.vector(age.rel[1:nan,23]) ### vector of age at release

### YEAR OF CAPTURE

year=as.matrix(read.csv("tortcr_year.csv",header=F)[1:nan,1:ns])

year=year[1:nan,1:ns]

year2 <- year[1,] # year corresponding to each survey bout in the capture history

year2[19] <- 1993

### convert actual year into a small integer with 1 representing the first year

realyears <- seq(1975,2007,1) # real years of the study

nyears <- length(realyears) # total years of the study period

numyear <- apply(year,c(1,2),function(t) which(t==realyears)) # convert to year of study

numyear2 <- numyear[1,] # vector: years of surveys, with "1" as baseline

### AGE

age=as.matrix(read.csv("tortcr_age.csv",header=F)[1:nan,1:ns])

age <- apply(age,c(1,2),function(t) ifelse(is.na(t),1,t))

### years to maturity, based on age

ytm <- apply(age,c(1,2),function(t) ifelse((8-t)<0,0,8-t)) ## years to "maturity", assuming adult survival is achieved at age 8

### PRECIPITATION

rain=as.matrix(read.csv("tortcr_rain.csv",header=F)[1:nan,1:ns])

rain=as.vector(rain[1,]) # vector of precip for each survey year

mean.rain=mean(rain,na.rm=T) # mean precip, for standardization

sd.rain=sd(as.vector(rain),na.rm=T) # sd precip, for standardization

### YEARS IN STUDY (binary, indicates whether the individual has been released (1) or has not yet been released (0))

z2 <- as.matrix(read.csv("tortcr_age.csv",header=F)[1:nan,1:ns])

Z <- apply(z2,c(1,2),function(t) ifelse(is.na(t),0,1) )

### NUMBERS OF RELEASED INDIVIDUALS AT TUNAS AND CACO

tot_release <- read.csv("tot_release.csv",header=T)

site_totrel <- tot_release$site

year_totrel <- tot_release$year[-which(site_totrel=="Gardner")]

age_totrel <- tot_release$age[-which(site_totrel=="Gardner")]

rel_totrel <- tot_release$released_notdata[-which(site_totrel=="Gardner")]

totrel_totrel <- tot_release$released[-which(site_totrel=="Gardner")]

### AND GARDNER

year_totrelg <- tot_release$year[which(site_totrel=="Gardner")]

age_totrelg <- tot_release$age[which(site_totrel=="Gardner")]

rel_totrelg <- tot_release$released_notdata[which(site_totrel=="Gardner")]

totrel_totrelg <- tot_release$released[which(site_totrel=="Gardner")]

### CAPTURE HISTORY INFO FOR GARDNER

caphistgg <- as.matrix(read.csv("g_caphist.csv",header=F))

agegg <- as.matrix(read.csv("g_age.csv",header=F))

agerelg <- as.vector(read.csv("g_rel.age.csv", header=F)[,1])

nang <- nrow(caphistgg)

nsg <- ncol(caphistgg)

yeargg=as.vector(c(1999,2000,2001,2002,2003,2007))

#######################################################

########First set of releases, 1975-1991 PRE-PIT TAGGING (branding, notching) (suffix "pre")

caphistppre<-read.csv(file="Master_capture.csv",header=FALSE)

caphistppre<-as.matrix(caphistppre)

nanpre=nrow(caphistppre)

nspre=ncol(caphistppre)

nyearspre=nspre

yearpre <- year2[1:nspre]

yrs.sincepre <- c(1,1,1,1,1,1,1,1,1,1,1,1,1,1,1,1)

ageppre<-read.csv("age.csv", header=FALSE)

ageppre<-as.matrix(ageppre)

ageppre <- apply(ageppre,c(1,2),function(t) ifelse(is.na(t),1,t))

#########################

# POST-pit tag capture history dataset...

caphistppost=read.csv("pit_caphist.csv",header=FALSE)

caphistppost=as.matrix(caphistppost)

nanpost=nrow(caphistppost)

nspost=ncol(caphistppost)

yearpost <- year2[(ns-nspost+1):ns]

yrs.sincepost=c(1,7,2,1,4)

age.relpost=read.csv("pit_age.csv",header=FALSE)

agerelpost=as.vector(age.relpost$V1)

agerelpost[149] = 5

#######################################

#Binary matrix "release" indicating year when animal was released (1 for release, 0 for all else)

first<-numeric(nan)

release<-matrix(0,nrow=nan,ncol=ns)

for(i in 1:nan){

for(j in 1:ns){

if(caphist[i,j]==1){

first[i]=j ### vector: year of first capture

release[i,j]=1 ### matrix: flag year of release

break}

}

}

releases <- apply(release,2,sum) # total released each year, based on original capture history

released <- numeric(ns)

released[1] <- releases[1]

for(t in 2:ns){

released[t] <- released[t-1] + releases[t] # cumulative sum of releases

}

##########################################

##### CONVERT MATRICES WITH ONLY SURVEY YEARS TO MATRICES WITH ALL YEARS... (suffix "b")

caphistb <- matrix(NA,nrow=nan,ncol=nyears) # recreate capture history, this time with all years

counter <- 1

for(t in numyear2){

caphistb[,t] <- caphist[,counter]

counter <- counter + 1

}

firstb<-numeric(nan)

releaseb<-matrix(0,nrow=nan,ncol=nyears)

for(i in 1:nan){

for(j in 1:nyears){

if(caphistb[i,j]==1&!is.na(caphistb[i,j])){

firstb[i]=j # vector of first captures

releaseb[i,j]=1 # matrix: flag release

break

}

}

}

ageb <- matrix(1,nrow=nan,ncol=nyears)

for(i in 1:nan){

for(t in firstb[i]:nyears){

ageb[i,t] <- agerel[i] + (t-firstb[i]) # matrix of age

}

}

ytmb <- apply(ageb,c(1,2),function(t) ifelse((8-t)<0,0,8-t)) # matrix of years to maturity

Z <- apply(ageb,c(1,2),function(t) ifelse(t==1,0,1) ) # matrix of post-release vs. pre-release

##########################################

############ SUMMARIZE ALL RELEASED ANIMALS... suffix "2"

nan2 <- sum(totrel_totrel) # total individuals released

yearrel_totrel <- rep(year_totrel,times=totrel_totrel) # vector: actual year of release for each individual

numyear_totrel <- as.vector(apply(as.matrix(yearrel_totrel),c(1,2),function(t) which(t==realyears))) # same as above: "1" as baseline

agerel_totrel <- rep(age_totrel,times=totrel_totrel) # vector: age at release for each individual

####### generate a "first2" dataset for these released animals

first2 <- numeric(nan2)

for(i in 1:nan2){

for(t in 1:nyears){

if(numyear_totrel[i]==t){

first2[i] <- t # vector of first "capture" in CJS terms, actually release event

break

}

}

}

####### generate matrix covariates for all releases

agemat_totrel <- matrix(1,nrow=nan2,ncol=nyears)

relmat_totrel <- matrix(0,nrow=nan2,ncol=nyears)

caphist2 <- matrix(NA,nrow=nan2,ncol=nyears) # final capture history

for(i in 1:nan2){

for(t in first2[i]:nyears){

if(t==first2[i]) relmat_totrel[i,t] <- 1 # matrix: flag release year for each t+c release

agemat_totrel[i,t] <- agerel_totrel[i] + (t-first2[i]) # matrix: age of each T+C release at each year of study

if(t==first2[i]) caphist2[i,t] <- 1 # master capture history should read "1" for release

}

}

#ytm_totrel <- apply(agemat_totrel,c(1,2),function(t) ifelse((8-t)<0,0,8-t))

###################

# DEAL WITH GARDNER DATA (suffix "g")

nan2g <- sum(totrel_totrelg)

yearrel_totrelg <- rep(year_totrelg,times=totrel_totrelg)

numyear_totrelg <- as.vector(apply(as.matrix(yearrel_totrelg),c(1,2),function(t) which(t==realyears)))

agerel_totrelg <- rep(age_totrelg,times=totrel_totrelg)

####### generate a "first2" dataset for these released animals

first2g <- numeric(nan2g)

for(i in 1:nan2g){

for(t in 1:nyears){

if(numyear_totrelg[i]==t){

first2g[i] <- t # vector: year of release at Gardner site

break

}

}

}

####### generate matrix covariates for all releases

agemat_totrelg <- matrix(1,nrow=nan2g,ncol=nyears)

relmat_totrelg <- matrix(0,nrow=nan2g,ncol=nyears)

caphist2g <- matrix(NA,nrow=nan2g,ncol=nyears)

for(i in 1:nan2g){

for(t in first2g[i]:nyears){

if(t==first2g[i]) relmat_totrelg[i,t] <- 1

agemat_totrelg[i,t] <- agerel_totrelg[i] + (t-first2g[i]) # matrix of tortoise age for all gardner releases

if(t==first2g[i]) caphist2g[i,t] <- 1 # gardner release site, capture history

}

}

### Manipulate gardner data

nan2g <- nrow(relmat_totrelg) # total number of individuals released at gardner

numyear2g <- which(realyears%in%yeargg)

caphistg <- matrix(0,nrow=nang,ncol=nyears)

counter <- 1

for(t in numyear2g){

caphistg[,t] <- caphistgg[,counter] ## original Gardner capture history, expanded to all years

counter <- counter + 1

}

firstg<-numeric(nang)

releaseg<-matrix(0,nrow=nang,ncol=nyears)

for(i in 1:nang){

for(j in 1:nyears){

if(caphistg[i,j]==1&!is.na(caphistg[i,j])){

firstg[i]=j # vector of first captures, gardner

releaseg[i,j]=1 # matrix flags release year for each individual

break

}

}

}

ageg <- matrix(1,nrow=nang,ncol=nyears)

for(i in 1:nang){

for(t in firstg[i]:nyears){

ageg[i,t] <- agerelg[i] + (t-firstg[i]) # age at each year, gardner

}

}

Zg <- apply(ageg,c(1,2),function(t) ifelse(t==1,0,1) ) # pre or post release

relyearsg <- apply(relmat_totrelg,2,function(t) ifelse(sum(t)>10,1,0)) # is it a release year or not

nrelsg <- sum(relyearsg) # total number of gardner releases

relindexg <- numeric(nyears)

relindexg[1] <- 0

for(t in 2:nyears){

relindexg[t]=relindexg[t-1]+relyearsg[t]

}

relindexg[nyears] <- relindexg[nyears-1]

relindexg[which(relindexg==0)] <- 1 # for each year, the cumulative number of releases to date

ramg <- releaseg*ageg # matrix: for each release, the age of the tortoise, "release age matrix", gardner

# note: for linking caphist information with release information...

############ LINK RELEASE INFORMATION, PRE-PITTAG INFORMATION, AND POST-PITTAG INFORMATION

caphistmasterg <- array(0,dim=c(nan2g,nyears))

indavailg <- rep(1,nang) # flag for whether each capture history row is still "up for grabs"

i=155; t=27

for(i in 1:nan2g){ # loop through total number of individuals released at gardner

for(t in 1:nyears){ # loop through total number of years

if(relmat_totrelg[i,t]==1){ # if the animal was just released...

if(length(which(ramg[,t]==agemat_totrelg[i,t]&indavailg==1))>0){ # find a capture history that matches with that release...

temp1 <- ifelse(length(which(ramg[,t]==agemat_totrelg[i,t]&indavailg==1))>1,

sample(which(ramg[,t]==agemat_totrelg[i,t]&indavailg==1),1), # sample randomly from available matches

which(ramg[,t]==agemat_totrelg[i,t]&indavailg==1) )

caphistmasterg[i,t:nyears] <- caphistg[temp1,t:nyears]

indavailg[temp1] <- 0

} else if(length(which(releaseg[,t]==1&indavailg==1))>0){

temp1 <- ifelse(length(which(releaseg[,t]==1&indavailg==1))>1,

sample(which(releaseg[,t]==1&indavailg==1),1),

which(releaseg[,t]==1&indavailg==1) )

caphistmasterg[i,t:nyears] <- caphistg[temp1,t:nyears]

indavailg[temp1] <- 0

} else if(length(which(releaseg[,(t-1)]==1&indavailg==1))>0){

temp1 <- ifelse(length(which(releaseg[,(t-1)]==1&indavailg==1))>1,

sample(which(releaseg[,(t-1)]==1&indavailg==1),1),

which(releaseg[,(t-1)]==1&indavailg==1) )

caphistmasterg[i,t:nyears] <- caphistg[temp1,t:nyears]

indavailg[temp1] <- 0

} else if(length(which(releaseg[,(t+1)]==1&indavailg==1))>0){

temp1 <- ifelse(length(which(releaseg[,(t+1)]==1&indavailg==1))>1,

sample(which(releaseg[,(t+1)]==1&indavailg==1),1),

which(releaseg[,(t+1)]==1&indavailg==1) )

caphistmasterg[i,t:nyears] <- caphistg[temp1,t:nyears]

indavailg[temp1] <- 0

} else if(length(which(releaseg[,(t-2)]==1&indavailg==1))>0){

temp1 <- ifelse(length(which(releaseg[,(t-2)]==1&indavailg==1))>1,

sample(which(releaseg[,(t-2)]==1&indavailg==1),1),

which(releaseg[,(t-2)]==1&indavailg==1) )

caphistmasterg[i,t:nyears] <- caphistg[temp1,t:nyears]

indavailg[temp1] <- 0

} else if(length(which(releaseg[,(t+2)]==1&indavailg==1))>0){

temp1 <- ifelse(length(which(releaseg[,(t+2)]==1&indavailg==1))>1,

sample(which(releaseg[,(t+2)]==1&indavailg==1),1),

which(releaseg[,(t+2)]==1&indavailg==1) )

caphistmasterg[i,t:nyears] <- caphistg[temp1,t:nyears]

indavailg[temp1] <- 0

}

}

}

}

caphistmasterg2<-caphistmasterg+relmat_totrelg # touch up the master capture history data set for Gardner

caphistmasterg2 <- apply((caphistmasterg+relmat_totrelg),c(1,2),function(t) ifelse(t>0,1,0))

#caphistmaster3 <- apply(caphistmaster,c(1,2) function(t) ifelse(t>0,1,0) )

survyearsg <- ifelse(apply(caphistg,2,sum)>0,1,0) # all "survey" years- must also include release years...

year2g <- realyears[which(survyearsg==1)]

nsurveysg <- length(year2g)

survindexg <- length(nyears)

counter <- 1

for(y in 1:nyears) {

if(realyears[y] == year2g[counter]) counter=counter+1

survindexg[y] <- counter # cumulative number of releases at Gardner to date

}

survindexg <- survindexg-1

survindexg[which(survindexg==0)] <- 1

#############################################################

###################################################

#### Deal with split datasets for tunas and caco- pre pit and post pit

#Binary variable "release" of year when animal was released

firstpost<-numeric(nanpost)

releasepost<-matrix(0,nrow=nanpost,ncol=nspost)

for(i in 1:nanpost){

for(j in 1:nspost){

if(caphistppost[i,j]==1){

firstpost[i]=j

releasepost[i,j]=1

break}

}

}

#Get linear age

ageppost=matrix(0,nrow=nanpost,ncol=nspost+1)

for (i in 1:nanpost){

for (j in 1:nspost){

if(caphistppost[i,j]==1){

ageppost[i,j]=age.relpost[i,1]

break}

}

}

for (i in 1:nanpost){

for (j in 1:nspost){

if(ageppost[i,j]!=0){

ageppost[i,j+1]=ageppost[i,j]+yrs.sincepost[j]

}

}

}

ageppost=ageppost[,1:6]

ageppost <- apply(ageppost,c(1,2),function(t) ifelse(t==0,1,t))

Zppost <- matrix(0,nrow=nanpost,ncol=nspost)

for(i in 1:nanpost){

for(j in 1:nspost){

if(releasepost[i,j]==1){

Zppost[i,j:nspost]=1

}

}

}

##############################################

### Manipulate post data

numyear2post <- which(realyears%in%yearpost)

caphistpost <- matrix(0,nrow=nanpost,ncol=nyears)

counter <- 1

for(t in numyear2post){

caphistpost[,t] <- caphistppost[,counter]

counter <- counter + 1

}

firstpost<-numeric(nanpost)

releasepost2<-matrix(0,nrow=nanpost,ncol=nyears)

for(i in 1:nanpost){

for(j in 1:nyears){

if(caphistpost[i,j]==1&!is.na(caphistpost[i,j])){

firstpost[i]=j

releasepost2[i,j]=1

break

}

}

}

lastpost<-numeric(nanpost)

for(i in 1:nanpost){

for(j in nyears:1){

if(caphistpost[i,j]==1&!is.na(caphistpost[i,j])){

lastpost[i]=j

break

}

}

}

agepost2 <- matrix(1,nrow=nanpost,ncol=nyears)

for(i in 1:nanpost){

for(t in 1:nyears){

agepost2[i,t] <- agerelpost[i] + (t-firstpost[i])

}

}

releasepost <- apply(agepost2,c(1,2),function(t) ifelse(t==4,1,0) )

Zpost <- apply(agepost2,c(1,2),function(t) ifelse(t==1,0,1) )

nyearspost=ncol(caphistpost)-nyearspre

########################

##### MANIPULATE PRE-PIT DATA (from tunas and caco)

#Binary variable "release" of year when animal was released

firstpre<-numeric(nanpre)

releasepre<-matrix(0,nrow=nanpre,ncol=nspre)

for(i in 1:nanpre){

for(j in 1:nspre){

if(caphistppre[i,j]==1){

firstpre[i]=j

releasepre[i,j]=1

break}

}

}

Zpre <- matrix(0,nrow=nanpre,ncol=nspre)

for(i in 1:nanpre){

for(j in 1:nspre){

if(releasepre[i,j]==1){

Zpre[i,j:nspre]=1

}

}

}

##############################################

### Manipulate pre data

numyear2pre <- which(realyears%in%yearpre)

caphistpre <- matrix(0,nrow=nanpre,ncol=nyears)

counter <- 1

for(t in numyear2pre){

caphistpre[,t] <- caphistppre[,counter]

counter <- counter + 1

}

firstpre<-numeric(nanpre)

releasepre<-matrix(0,nrow=nanpre,ncol=nyears)

for(i in 1:nanpre){

for(j in 1:nyears){

if(caphistpre[i,j]==1&!is.na(caphistpre[i,j])){

firstpre[i]=j

releasepre[i,j]=1

break

}

}

}

lastpre<-numeric(nanpre)

for(i in 1:nanpre){

for(j in nyears:1){

if(caphistpre[i,j]==1&!is.na(caphistpre[i,j])){

lastpre[i]=j

break

}

}

}

agepre <- matrix(1,nrow=nanpre,ncol=nyears)

for(i in 1:nanpre){

for(t in firstpre[i]:nyearspre){

agepre[i,t] <- ageppre[i,t]

}

}

Zpre <- apply(agepre,c(1,2),function(t) ifelse(t==1,0,1) )

survyears<-as.vector(apply(as.matrix(realyears),1,function(t) ifelse(t%in%year2,1,0))) # survey years...

yrs.since[19:20] <- c(1,7)

prevsurv <- c(as.vector(rep(year2[1:(ns-1)],yrs.since[2:ns])),2007)

prevsurv2 <- as.vector(apply(as.matrix(prevsurv),1,function(t) which(realyears==t)))

caphistpre <- caphistpre-releasepre

#############################################################

############# BUILD MASTER DATASET: TUNAS AND CACO

############ Build a master dataset that includes all releases on T+C

# key inputs: agemat_totrel, relmat_totrel

# generate age class variables...

ageclassfunc<- function(age){

ifelse(age<2,0,ifelse(age<4,1,ifelse(age<7,2,ifelse(age<10,3,ifelse(age<15,4,5))))) # convert age to age class (for combining data sets)

}

nagecl <- 5

ageclassfunc2<- function(age){

ifelse(age<2,0,ifelse(age<7,1,ifelse(age<15,2,3)))

}

nagecl2 <- 3

agecl_totrel <- apply(agemat_totrel,c(1,2), ageclassfunc )

ageclpost2 <- apply(agepost2,c(1,2), ageclassfunc2 )

ageclpre <- apply(agepre,c(1,2), ageclassfunc )

rampre <- releasepre*agepre

ramclpre <- apply(rampre,c(1,2),ageclassfunc)

# find the capture histories in the pre-pit dataset that don't line up with the release dataset...

####### revise the post-pit age and age class variables...

numrel <- apply(relmat_totrel,2,sum)

avgagerel <- apply(relmat_totrel*agemat_totrel,2,sum) / numrel

avgagerel[which(is.nan(avgagerel))] <- 0

agerel1 <- ifelse(avgagerel==0, -100,floor(avgagerel))

agerel2 <- ifelse(avgagerel==0, -100,ceiling(avgagerel))

agerel.per1 <- 1-(avgagerel-agerel1)

agefunc1 <- function(agecl,firstyear){ ## function for linking data sets on the basis of inferred age...

poss.ages <- list() ## returns inferred age class for year of switch to pit tags...

poss.ages[[1]] <- c(4:6)

poss.ages[[2]] <- c(7:14)

poss.ages[[3]] <- c(15:40)

surv <- c(.8,.9,.95)

length <- length(c(1:firstyear))

possyears <- c(1:firstyear)

prob1 <- numeric(length)

prob2 <- numeric(length)

counter <- 0

for(t in firstyear:1){

prob1[t] <- surv[agecl]^counter

pa2 <- poss.ages[[agecl]] - counter

if(agerel1[t]%in%pa2){

prob2[t] <- prob2[t] + prob1[t]*numavail[t]*agerel.per1[t]

}

if(agerel2[t]%in%pa2){

prob2[t] <- prob2[t] + prob1[t]*numavail[t]*(1-agerel.per1[t])

}

if(length(intersect(c(agerel1[t],agerel2[t]),pa2))==0){

prob2[t] <- ifelse(numrel[t]>0,0.01 * prob1[t],0)

}

counter <- counter+1

}

realyear <- sample(possyears,1,prob=prob2)

realagerel <- sample(c(agerel1[realyear],agerel2[realyear]),1,prob=c(agerel.per1[realyear],(1-agerel.per1[realyear])))

realagefirst <- realagerel + (firstyear-realyear)

return(c(realagefirst,realyear,realagefirst))

}

agepost <- array(0,dim=c(nanpost,nyears) )

releasepost <- array(0,dim=c(nanpost,nyears) )

numavail <- numrel

for(i in 1:nanpost){

temp1 <- agefunc1(ageclpost2[i,firstpost[i]],firstpost[i])

agepost3 <- temp1[1]

temp2 <- temp1[2]

for(t in 1:nyears){

agepost[i,t] <- agepost3 + (t-firstpost[i])

if(t == temp2) releasepost[i,t] <- 1

if(t == temp2) numavail[t] <- numavail[t] - 1

}

}

pos.rel <- apply(releasepost,2,sum)

rbind(numrel,pos.rel)

ageclpost <- apply(agepost,c(1,2), ageclassfunc )

rampost <- releasepost*agepost

ramclpost <- apply(rampost,c(1,2),ageclassfunc)

# set variables

is.gard <- numeric(nan2)

is.tc <- numeric(nan2)

last2 <- numeric(nan2)

caphistmaster <- array(0,dim=c(nan2,nyears))

indavailpre <- rep(1,nanpre)

indavailpost <- rep(1,nanpost)

#ncaptures1992 <-

#avail1992 <-

for(i in 1:nan2){ # loop through individuals in the master release dataset

for(t in 1:nyears){ # loop through years in the master release dataset

if(relmat_totrel[i,t]==1){ # if the animal was just released and in the pre-pit time frame...

if(t<=nyearspre){

if(length(which(rampre[,t]==agemat_totrel[i,t]&indavailpre==1))>0){ # find a capture history that fits with that release...

temp1 <- ifelse(length(which(rampre[,t]==agemat_totrel[i,t]&indavailpre==1))>1,

sample(which(rampre[,t]==agemat_totrel[i,t]&indavailpre==1),1),

which(rampre[,t]==agemat_totrel[i,t]&indavailpre==1) )

caphistmaster[i,t:nyearspre] <- caphistpre[temp1,t:nyearspre]

indavailpre[temp1] <- 0

last2[i] <- lastpre[temp1] # last time it was captured...

} else if(length(which(releasepre[,t]==1&indavailpre==1))>0){

temp1 <- ifelse(length(which(releasepre[,t]==1&indavailpre==1))>1,

sample(which(releasepre[,t]==1&indavailpre==1),1),

which(releasepre[,t]==1&indavailpre==1) )

caphistmaster[i,t:nyearspre] <- caphistpre[temp1,t:nyearspre]

indavailpre[temp1] <- 0

last2[i] <- lastpre[temp1]

} else if(length(which(releasepre[,(t-1)]==1&indavailpre==1))>0){

temp1 <- ifelse(length(which(releasepre[,(t-1)]==1&indavailpre==1))>1,

sample(which(releasepre[,(t-1)]==1&indavailpre==1),1),

which(releasepre[,(t-1)]==1&indavailpre==1) )

caphistmaster[i,t:nyearspre] <- caphistpre[temp1,t:nyearspre]

indavailpre[temp1] <- 0

last2[i] <- lastpre[temp1]

} else if(length(which(releasepre[,(t+1)]==1&indavailpre==1))>0){

temp1 <- ifelse(length(which(releasepre[,(t+1)]==1&indavailpre==1))>1,

sample(which(releasepre[,(t+1)]==1&indavailpre==1),1),

which(releasepre[,(t+1)]==1&indavailpre==1) )

caphistmaster[i,t:nyearspre] <- caphistpre[temp1,t:nyearspre]

indavailpre[temp1] <- 0

last2[i] <- lastpre[temp1]

}

}

}

}

}

usedreleases <- numeric(nyears)

for(i in 1:nan2){ # fill in dataset for those animals released post-pit...

for(t in (nyearspre+1):nyears){

if(relmat_totrel[i,t]==1){

if(t>nyearspre&t<=nyears){

if(length(which(rampost[,t]==agemat_totrel[i,t]&indavailpost==1))>0){

temp2 <- ifelse(length(which(rampost[,t]==agemat_totrel[i,t]&indavailpost==1))>1,

sample(which(rampost[,t]==agemat_totrel[i,t]&indavailpost==1),1),

which(rampost[,t]==agemat_totrel[i,t]&indavailpost==1) )

caphistmaster[i,t:nyears] <- caphistpost[temp2,t:nyears]

indavailpost[temp2] <- 0

usedreleases[t] <- usedreleases[t] + 1

}

}

}

}

}

cbind(numrel[18:33],usedreleases[18:33])

# link the remaining unassigned post-pit individuals to a pre-pit individual...

# match up the pre-pit and post-pit datasets, conservatively...

# loop through the remaining individuals, and assign them each to a pre-pit individual

# according to its most-recent capture date.

whichavailpre <- list()

totavailpre <- list() #

whichavailpost <- list()

temp3 <- array(0,dim=c(nyearspre,max(numrel[1:nyearspre])))

for(t in 1:nyearspre){

counter <- 1

if(numrel[t]>0){

whichavailpre[[t]] <- which(relmat_totrel[,t]==1) # all pre-pit releases at age class a

totavailpre[[t]] <- length(whichavailpre[[t]]) #

whichavailpost[[t]] <- which(indavailpost==1&releasepost[,t]==1)

for(i in whichavailpost[[t]]){

if(totavailpre[[t]]>0){

temp3[t,counter] <- ifelse(totavailpre[[t]]>1,

sample(whichavailpre[[t]],prob=(last2[whichavailpre[[t]]]-min(last2[whichavailpre[[t]]])+1),1),

whichavailpre[[t]])

caphistmaster[temp3[t,counter],(nyearspre+1):nyears] <- caphistpost[i,(nyearspre+1):nyears]

indavailpost[i] <- 0

whichavailpre[[t]] <- whichavailpre[[t]][-which(whichavailpre[[t]]==temp3[t,counter])]

totavailpre[[t]] <- length(whichavailpre[[t]])

counter <- counter+1

}

}

}

}

caphistpost[which(indavailpost==1),18:33]*agepost[which(indavailpost==1),18:33]

caphistmaster2<-caphistmaster+relmat_totrel

caphistmaster2[which(relmat_totrel[,1]==1),]

caphistmaster2 <- apply((caphistmaster+relmat_totrel),c(1,2),function(t) ifelse(t>0,1,0))

#caphistmaster3 <- apply(caphistmaster,c(1,2) function(t) ifelse(t>0,1,0) )

caphistmaster3 <- caphistmaster2 - relmat_totrel

agemat_totrel2 <- apply(agemat_totrel,c(1,2),function(t) ifelse(t==1,0,t))

survyears3_1 <- which(realyears%in%year2)

survyears3 <- c(rep(survyears3_1[1:22],times=yrs.since[2:23]),rep(survyears3_1[23],times=1))

survindex<-as.numeric(as.factor(survyears3))

nsurveys <- length(unique(survyears3))

###############

# COMPUTE A "PERIOD" VARIABLE

period <- rep(c(1,2,3,4),times=c(10,4,4,15)) # adult survivorship index

nperiods <- max(period)

period2 <- rep(c(1:11),times=c(2,2,2,2,2,2,2,2,2,9,6)) # adult survivorship index

nperiods2 <- max(period2)

relyears <- apply(relmat_totrel,2,function(t) ifelse(sum(t)>10,1,0))

nrels <- sum(relyears)-1

relindex <- numeric(nyears)

relindex[1] <- 1

for(t in 2:nyears){

relindex[t]=relindex[t-1]+relyears[t]

}

relindex[nyears] <- relindex[nyears-1]

##################################################################

#################################################### RUN WINBUGS FOR PARAMETER ESTIMATION

Z2 <- apply(agemat_totrel,c(1,2),function(t) ifelse(t>1,1,0))

Z2g <- apply(agemat_totrelg,c(1,2),function(t) ifelse(t>1,1,0))

Data <- list(

y = caphistmaster2,

yg = caphistmasterg2,

is.rel = relmat_totrel,

is.relg = relmat_totrelg,

nan = nan2,

nang = nan2g,

nyears = nyears,

age2 = agemat_totrel2,

age2g = agemat_totrelg,

first = first2,

firstg = first2g,

period = period,

nperiods = nperiods,

period2 = period2,

nperiods2 = nperiods2,

survyears = survyears,

survindex = survindex,

survyearsg = survyearsg,

survindexg = survindexg,

nsurveys = nsurveys,

nsurveysg = nsurveysg,

relindex = relindex,

relindexg = relindexg,

nrels = nrels,

nrelsg = nrelsg

)

Inits <- function() list( # initial values for all stochastic nodes.

phijuv = runif(nperiods2, 0.95, 0.97),

phiadult = runif(nperiods, 0.95, 0.97),

adultbeta_p = runif(1,-.05,.05),

p0 = runif(nsurveys, 0.3, 0.4),

relbeta_phi = runif(1,-.5,-0.25),

alive = Z2,

aliveg = Z2g

)

Par <- c("phiadult",

"phijuv",

"adultbeta_p",

"p0",

"p0g",

"Na",

"Nj",

"relbeta_phi",

"sd.phijuv",

"sd.phiadult",

"N",

"Ng",

"Nag",

"Njg",

"meanjuv",

"meanadult",

"p_badyr",

"badyr",

"badyrg"

)

BugFile <- ("espCJS10_norobust1_KTS9_works7.bug")

Mod <- bugs(data=Data, inits=Inits, parameters.to.save=Par, # run BUGS with 3 chains

model.file=BugFile, n.chains=1, n.iter=20000,

bugs.directory="C:/Users/grads/Desktop/winbugs14/WinBUGS14",

n.burnin=10000,n.thin=5,debug=T) # codaPkg=TRUE,

# "C:/Users/grads/Desktop/winbugs14/WinBUGS14",

# "C:/Users/Kevin/Documents/Academic/Bog Turtle/DATA/software/BUGS/WinBUGS14",

## WinBUGS code

##########################################

# BEGIN MODEL

model {

###############################################

# SET PRIORS

for(t in 1:nperiods){

phiadult[t] ~ dunif(.001,.999)

logit.phiadult[t] <- log(phiadult[t]/(1-phiadult[t]))

}

for(t in 1:nperiods2){

phijuv[t] ~ dunif(.001,.999)

logit.phijuv[t] <- log(phijuv[t]/(1-phijuv[t]))

}

p0[1] <- 0.999999

logit.p[1] <- log(p0[1]/(1-p0[1]))

for(t in 2:nsurveys){

p0[t] ~ dunif(.001,.999) # mean probability of capture (prior)

logit.p[t] <- log(p0[t]/(1-p0[t])) # prior for average capture probability per (sub)survey occasion

}

p0g[1] <- 0.99999

logit.pg[1] <- log(p0g[1]/(1-p0g[1]))

for(t in 2:nsurveysg){

p0g[t] ~ dunif(.001,.8) # mean probability of capture (prior)

logit.pg[t] <- log(p0g[t]/(1-p0g[t])) # prior for average capture probability per (sub)survey occasion

}

pgoodyr[1] <- 1/3

pgoodyr[2] <- 1/3

pgoodyr[3] <- 1/3

goodyr2[1] <- 0

goodyr2[2] <- 1

goodyr2[3] <- 2

for(t in 1:nrels){

goodyr[t] ~ dcat(pgoodyr[])

badyr[t] <- goodyr2[goodyr[t]]

}

for(t in 1:nrelsg){

goodyrg[t] ~ dcat(pgoodyr[])

badyrg[t] <- goodyr2[goodyrg[t]]

}

relbeta_phi ~ dunif(-6,-0.5) # prior for effect of survival after release

adultbeta_p ~ dunif(-5,5)

##########################################################

# INITIALIZE MATRICES FOR PRE-RELEASE

# for calculating pop. size at Tunas+Caco and Gardiner

for(i in 1:nan){

for(t in 1:(first[i]-1)){

alive[i,t] <- 0

}

}

for(i in 1:nang){

for(t in 1:(firstg[i]-1)){

aliveg[i,t] <- 0

}

}

##########################################################

### PROCESS MODEL

############ set covariates.

for(i in 1:nan){

for(t in 1:nyears){

is.adult2[i,t] <- step(age2[i,t]-8) # adult survival starting at age 8

is.juv2[i,t] <- step(7-age2[i,t])

is.juv[i,t] <- step(14-age2[i,t])

is.adult[i,t] <- step(age2[i,t]-15)

}

}

############ set SURVIVAL submodel

for(i in 1:nan){

for(t in 1:(nyears-1)){

basesurv[i,t] <- logit.phijuv[period2[t]]*is.juv2[i,t] + logit.phiadult[period[t]]*is.adult2[i,t]

logit(phi[i,t]) <- basesurv[i,t] + relbeta_phi * badyr[relindex[t]] * is.rel[i,t]

}

}

########## process model

for(i in 1:nan){

alive[i,first[i]] ~ dbern(1)

for(t in (first[i]+1):nyears){

mualive[i,t] <- alive[i,(t-1)] * phi[i,(t-1)]

alive[i,t] ~ dbern(mualive[i,t]) # latent variable- still alive

}

}

####################################

# OBSERVATION MODEL

for(i in 1:nan) {

y[i,first[i]] ~ dbern(1)

for(t in (first[i]+1):nyears) {

logit(p[i,t]) <- logit.p[survindex[t]] + adultbeta_p*is.adult2[i,t]

mup[i,t] <- alive[i,t]*p[i,t]*survyears[t]

y[i,t] ~ dbern(mup[i,t]) # likelihood of observed data...

}

}

######################################################

### GARDINER RELEASE SITE

############ set covariates.

for(i in 1:nang){

for(t in 1:nyears){

is.adult2g[i,t] <- step(age2g[i,t]-8) # adult survival

is.juv2g[i,t] <- step(7-age2g[i,t])

is.juvg[i,t] <- step(14-age2g[i,t])

is.adultg[i,t] <- step(age2g[i,t]-15)

}

}

############ set SURVIVAL submodel

for(i in 1:nang){

for(t in 1:(nyears-1)){

basesurvg[i,t] <- logit.phijuv[period2[t]]*is.juv2g[i,t] + logit.phiadult[period[t]]*is.adult2g[i,t]

logit(phig[i,t]) <- basesurvg[i,t] + relbeta_phi * badyrg[relindexg[t]] * is.relg[i,t]

}

}

########## process model

for(i in 1:nang){

aliveg[i,firstg[i]] ~ dbern(1)

for(t in (firstg[i]+1):nyears){

mualiveg[i,t] <- aliveg[i,(t-1)] * phig[i,(t-1)]

aliveg[i,t] ~ dbern(mualiveg[i,t]) # latent variable- still alive

}

}

for(i in 1:nang) {

yg[i,firstg[i]] ~ dbern(1)

for(t in (firstg[i]+1):nyears) {

logit(pg[i,t]) <- logit.pg[survindexg[t]] + adultbeta_p*is.adult2g[i,t]

mupg[i,t] <- aliveg[i,t]*pg[i,t]*survyearsg[t]

yg[i,t] ~ dbern(mupg[i,t]) # likelihood of observed data...

}

}

######################################################

# calculate abundance

for(t in 1:nyears){

N[t] <- sum(alive[1:nan,t])

Na[t] <- inprod(alive[1:nan,t],is.adult[1:nan,t])

Nj[t] <- inprod(alive[1:nan,t],is.juv[1:nan,t])

Ng[t] <- sum(aliveg[1:nang,t])

Nag[t] <- inprod(aliveg[1:nang,t],is.adultg[1:nang,t])

Njg[t] <- inprod(aliveg[1:nang,t],is.juvg[1:nang,t])

}

###################################################

# calculate the expected survival for each age class up to age 15 (not considering release effect)

meanadult <- mean(logit.phiadult[2:nperiods])

meanjuv <- mean(logit.phijuv[2:nperiods2])

#process variance on logit scale

for(t in 2:nperiods){

temp1adult[t] <- pow((meanadult-logit.phiadult[t]),2)

}

for(t in 2:nperiods2){

temp1juv[t] <- pow((meanjuv-logit.phijuv[t]),2)

}

temp2juv <- sum(temp1juv[2:nperiods2]) / (nperiods2-1)

temp2adult <- sum(temp1adult[2:nperiods]) / (nperiods-1)

sd.phijuv <- sqrt(temp2juv)

sd.phiadult <- sqrt(temp2adult) #standard deviation in survival (logit) among years.

# probability of a bad release year... (tunas and caco)

p_badyr <- sum(badyr[1:nrels])/nrels

##########################################

} # END MODEL

##########################################
