# Supplementary material for: Demographic Outcomes and Ecosystem Implications of Giant Tortoise Reintroduction to Española Island, Galapagos
Source: PLoS One. 2014 Oct 28;9(10):e110742. doi: 10.1371/journal.pone.0110742 (PMC4211691; doi:10.1371/journal.pone.0110742)
Supplement: Appendix S3 — Contains R code for projecting population dynamics for the in situ population of Española giant tortoises. (DOCX) [file pone.0110742.s003.docx]

Gibbs, J.P. et al. Giant Tortoise Reintroduction to Española Island, Galápagos: Demographic Outcomes and Ecosystem Implications.

# Appendix S3: R code for projecting population dynamics for the Española giant tortoise population

##################

## SIMULATION OF ESPANOLA TORTOISE POPULATION

library(coda)

library(MASS)

library(R2WinBUGS)

####################################

# Set data directory

#####################################

# Read in the coda textfiles...

# read in binary coda datafile

load("Mod3")

summary(Mod2)

MCMC <- rbind(as.data.frame(Mod2[[1]]))

# read in the K data

load("K_Results_Coda.RData")

summary(k.nbinom.coda)

K_MCMC <- rbind(as.data.frame(k.nbinom.coda[[1]]),as.data.frame(k.nbinom.coda[[2]]),as.data.frame(k.nbinom.coda[[3]]))

##########################

# SET OVERALL PARAMETERS

nMCMC <- length(MCMC$'N[1]')

nperiods <- 4

nperiods2 <- 11

nrels <- 19

###########################

### POP PARAMS

nages <- 18

adultage <- 18

atm_surv <- 8

atm_rep <- 17

releff_breakpoint <- 13

initfracfem <- 0.5

##########################

# thin the K data frame

temp <- nrow(K_MCMC)

ndx <- sample(c(1:nrow(K_MCMC)),size=nMCMC, replace=F)

K_MCMC <- K_MCMC[ndx,]

############################

### SET FUNCTIONS

start.abundance <- function(r) {

a <- c( 0,0,0,0,0,0,0,rep(floor((MCMC$'Nj[33]'[r]+MCMC$'Njg[33]'[r])/8),times=8),0,rep(floor((MCMC$'Na[33]'[r]+MCMC$'Nag[33]'[r])/2),times=2) )

b <- rbinom(length(a),a,initfracfem)

return(b)

}

jsurvival <- function(r,p2) {

eval(parse(text=paste("MCMC$'phijuv[",p2,"]'",sep="")))[r]

}

asurvival <- function(r,p) {

eval(parse(text=paste("MCMC$'phiadult[",p,"]'",sep="")))[r]

}

releff <- function(r,nr) eval(parse(text=paste("MCMC$'relbeta_phi'",sep="")))[r]

ys_samp <- sample(seq(.6,.9,length=100),nMCMC,replace=T)

youngsurv <- function(r) ys_samp[r] #seq(.6,.9,length=nMCMC)[r]

K <- function(r){

eval(parse(text=paste("K_MCMC$'K'",sep="")))[r]

}

ff_samp <- sample(seq(.35,.65,length=100),nMCMC,replace=T)

fracfem <- function(r) ff_samp[r] #seq(0.35,0.65,length=nMCMC)[r] # female fraction

survivorship <- function(r,N,p,p2) {

temp <- c( rep(youngsurv(r),times=4), rep(jsurvival(r,p2),times=(atm_surv-5)),

rep(asurvival(r,p),times=(nages-atm_surv+1)) )

vector <- numeric(nages)

vector2 <- numeric(nages)

vector3 <- numeric(nages)

for(a in 1:nages){

vector[a] <- temp[a]

# model density dependence...

vector2[a] <- ifelse(a<atm_surv&N>(K(r)*.75), plogis(qlogis(vector[a])-.25-.5*(N/K(r)) ) , vector[a] ) # 1-N/K

vector3[a] <- ifelse(vector2[a]<=0,0.1,vector2[a])

}

return(vector3)

}

badyear <- function(r,ri) {

eval(parse(text=paste("MCMC$'badyr[",ri,"]'",sep="")))[r]

}

survrel <- function(r,s,by){

effr <- releff(r)

ifelse(by>0,plogis(qlogis(s)+by*effr),s)

}

cs_samp <- sample(seq(5,7,length=100),nMCMC,replace=T)

meanclutchsize <- function(r) cs_samp[r] # mean clutch size:

# comes from approx likelihood exercise... In crianza, hatching rate is about 37%

ev_samp <- sample(seq(.222,.293,length=100),nMCMC,replace=T)

meaneggviab <- function(r) ev_samp[r] #seq(.152,.257,length=nMCMC)[r]

hs_samp <- sample(seq(.222,.293,length=100),nMCMC,replace=T)

meanhatchsurv <- function(r,N) { #temp <- seq(.222,.293,length=nMCMC)[r]

temp <- hs_samp[r]

temp2<- ifelse(N>K(r),plogis(qlogis(temp)-1-(N/K(r))),temp)

return(temp2)

}

fertility <- function(r,N) {

r2 <- sample(c(1:nMCMC),1,replace=T)

temp1 <- meanclutchsize(r)*fracfem(r2) * meaneggviab(r) * meanhatchsurv(r,N) # number of female eggs produced...

temp2 <- ifelse(temp1<=0,0.05,temp1)

return(temp2)

}

totmass <- function(popvec,ysim){ # calculate mass in kg

temp <- numeric(nages)

for(age in 1:(adultage-1)){

temp[age] <- (0.127 * (85 * (1 - exp(-0.13 * age - 0.195))^1.85)^2.95 )/1000 * popvec[age]

}

temp[adultage] <- (0.127 * (85 * (1 - exp(-0.13 * (17+ysim) - 0.195))^1.85)^2.95 )/1000*popvec[age]

return(sum(temp))

}

#########################################################################

########################## ESPANOLA FUTURE SCENARIO: 150 YEARS, under 3 management scenarios

### SIMULATION SCENARIOS- remove tortoises for repatriating pinta? continue releasing turtles from crianza?

### funnel crianza turtles to Pinta?

nscenarios <- 3

nsims <- 1000

nyears <- 100

# release turtles from crianza for 25 years longer

numrel <- c(rep(50,times=25),rep(0,times=nyears-25))

agerel <- rep(5,times=nyears)

#####################

# START SIMULATION - ESPANOLA

# note: optimistic scenario in which survival of released individuals are sampled from observed distribution of good

# and bad years

# set up storage arrays.

N <- array(0,dim=c(nscenarios,nsims,nages,nyears))

impact <- array(0,dim=c(nscenarios,nsims,nyears))

# s=1;i=1

for(s in 1:nscenarios){

for(i in 1:nsims){

randnum <- sample(c(1:nMCMC),1)

period <- sample(c(1:nperiods),1)

period2 <- sample(c(1:nperiods2),1)

N[s,i,,1] <- start.abundance(randnum)

relindex <- sample(c(1:nrels),1)

impact[s,i,1] <- totmass(N[s,i,,1],1)

by <- badyear(randnum,relindex)

# y=2

for(y in 2:nyears){

if(y%%4==0) period <- sample(c(1:nperiods),1)

if(y%%2==0) period2 <- sample(c(1:nperiods2),1)

relindex <- sample(c(1:nrels),1) #ifelse(sum(N[s,i,2:adultage,y-1])<(K(randnum)/3),sample(c(1:releff_breakpoint),1),sample(c((releff_breakpoint+1):nrels),1))

by <- badyear(randnum,relindex)

surv <- survivorship(randnum,sum(N[s,i,2:adultage,y-1]),period,period2)

for(a in 2:atm_rep){

N[s,i,a,y] <- rbinom(1,N[s,i,a-1,y-1],surv[a-1])

}

N[s,i,adultage,y] <- rbinom(1,(N[s,i,adultage,y-1]),surv[adultage]) + rbinom(1,N[s,i,atm_rep,y-1],surv[atm_rep])

impact[s,i,y] <- totmass(N[s,i,,y],y)

Eoffspring <- N[s,i,adultage,y-1]*fertility(randnum,sum(N[s,i,2:adultage,y-1]))

N[s,i,1,y] <- rpois(1,Eoffspring)

# model releases

if(s==1) N[s,i,agerel[y]+1,y] <- N[s,i,agerel[y]+1,y] + rbinom(1,numrel[y-1],survrel(randnum,surv[agerel[y-1]],by))

if(s==3&y==2) N[s,i,adultage,y] <- N[s,i,adultage,y] - 50

}

}

}

#############################################################################################################

########################### REPATRIATE ESPANOLA, KEEP TRACK OF "NATIVOS"

# GOAL- what proportion "nativo" would we expect to see under various recruitment models

# Compare to the observed number of 18% in 2010, 36 years after the initiation of the

# Espanola repatriation program.

### SIMULATION SCENARIOS

nscenarios <- 10 # alter egg viability and hatchling survivorship to compare

# with observed numbers of "nativos" and get a crude estimate of fertility...

nsims <- 300

nyears <- 36

# alter the basic model parameters for this analysis....

lbev <- seq(0.08,0.4,length=nscenarios) #egg viability

lbhs <- seq(0.08,0.4,length=nscenarios) #hatchling survivorship

meaneggviab <- function(r) seq(lbev[s],lbev[s],length=nMCMC)[r]

meanhatchsurv <- function(r,N) {

temp <- seq(lbhs[s],lbhs[s],length=nMCMC)[r]

temp2<- ifelse(N>K(r),0.1,temp)

return(temp2)

}

########################################################

# read in the release data from a separate file

realyear <- c(1975:2010)

tot_release <- read.csv("tot_release.csv",header=T)

tr_year <- tot_release$year

tr_year2 <- unique(tr_year)

tr_release <- tot_release$released

tr_release2 <- as.vector(tapply(tr_release,tr_year,sum))

tr_age <- tot_release$age

tr_age2 <- round(as.vector(tapply(tr_age,tr_year,mean)),0)

numrel <- numeric(length(realyear))

agerel <- numeric(length(realyear))

counter <- 1

for(y in realyear){

if(y %in% tr_year) numrel[counter] <- tr_release2[which(tr_year2==y)]

if(y %in% tr_year) agerel[counter] <- tr_age2[which(tr_year2==y)]

counter <- counter + 1

}

# release turtles from crianza

#####################

# START SIMULATION

N <- array(0,dim=c(nscenarios,nsims,nages,nyears))

#impact <- array(0,dim=c(nscenarios,nsims,nyears))

nativo <- array(0,dim=c(nscenarios,nsims,nages,nyears))

fracnativo36 <- array(0,dim=c(nscenarios,nsims))

for(s in 1:nscenarios){

for(i in 1:nsims){

randnum <- sample(c(1:nMCMC),1)

period <- 1

period2 <- 1

relindex <- 1

N[s,i,,1] <- rep(0,times=nages) #(randnum)

nativo[s,i,,1] <- rep(0,times=nages)

#impact[s,i,1] <- totmass(N[s,i,,1],1)

by <- badyear(randnum,relindex)

for(y in 2:nyears){

period <- ifelse(y%%4==0,sample(c(1:nperiods),1),period)

period2 <- ifelse(period2<nperiods2, ifelse(y%%2==0,period2+1,period2), sample(c(1:nperiods2),1) )

if(relindex<nrels) relindex <- ifelse(numrel[y]>10,relindex+1,relindex)

if(relindex>=nrels) relindex <- sample(c(1:nrels),1)

by <- badyear(randnum, relindex)

surv <- survivorship(randnum,sum(N[s,i,2:adultage,y-1]),period,period2)

for(a in 2:atm_rep){

N[s,i,a,y] <- rbinom(1,N[s,i,a-1,y-1],surv[a-1])

nativo[s,i,a,y] <- rbinom(1,nativo[s,i,a-1,y-1],surv[a-1])

}

N[s,i,adultage,y] <- rbinom(1,(N[s,i,adultage,y-1]),surv[adultage]) + rbinom(1,N[s,i,atm_rep,y-1],surv[atm_rep])

nativo[s,i,adultage,y] <- rbinom(1,(nativo[s,i,adultage,y-1]),surv[adultage]) + rbinom(1,nativo[s,i,atm_rep,y-1],surv[atm_rep])

#impact[s,i,y] <- totmass(N[s,i,,y],y)

Eoffspring <- N[s,i,adultage,y-1]*fertility(randnum,sum(N[s,i,2:adultage,y-1]))

N[s,i,1,y] <- rpois(1,Eoffspring)

nativo[s,i,1,y] <- N[s,i,1,y]

# model releases

N[s,i,agerel[y]+1,y] <- N[s,i,agerel[y]+1,y] + rbinom(1,numrel[y-1],survrel(randnum,surv[5],by))

}

fracnativo36[s,i] <- sum(nativo[s,i,3:adultage,nyears])/sum(N[s,i,3:adultage,nyears])

}

}

obsrecyear <- c(1991,1992,1993,1994,2000,2002,2003,2007,2010)

obsrecpts <- c(0.015,0.02,0.025,0.026,0.03,0.06,0.07,0.09,0.17)

scenarios <- c(1:10)
